# The impact of modifier genes on cone-rod dystrophy heterogeneity: An explorative familial pilot study and a hypothesis on neurotransmission impairment

**DOI:** 10.1371/journal.pone.0278857

**Published:** 2022-12-09

**Authors:** Luigi Donato, Simona Alibrandi, Concetta Scimone, Carmela Rinaldi, Angela Dascola, Alessandro Calamuneri, Rosalia D’Angelo, Antonina Sidoti

**Affiliations:** 1 Division of Medical Biotechnologies and Preventive Medicine, Department of Biomedical and Dental Sciences and Morphofunctional Imaging, University of Messina, Messina, Italy; 2 Department of Biomolecular Strategies, Genetics, Cutting-Edge Therapies, I.E.ME.S.T., Palermo, Italy; 3 Department of Chemical, Biological, Pharmaceutical and Environmental Sciences, University of Messina, Messina, Italy; Carl von Ossietzky Universitat Oldenburg, GERMANY

## Abstract

Cone-rod dystrophies (CORDs) are a heterogeneous group of inherited retinopathies (IRDs) with more than 30 already known disease-causing genes. Uncertain phenotypes and extended range of intra- and interfamilial heterogenicity make still difficult to determine a precise genotype-phenotype correlation. Here, we used a next-generation sequencing approach to study a Sicilian family with a suspected form of CORD. Affected family members underwent ophthalmological examinations and a proband, blind from 50 years, underwent whole genome and exome sequencing. Variant analysis was enriched by pathway analysis and relevant variants were, then, investigated in other family members and in 100 healthy controls from Messina. CORD diagnosis with an intricate pattern of symptoms was confirmed by ophthalmological examinations. A total of about 50,000 variants were identified in both proband’s genome and exome. All affected family members presented specific genotypes mainly determined by mutated *GUCY2D* gene, and different phenotypical traits, mainly related to focus and color perception. Thus, we looked for possible modifier genes. According to relationship with *GUCY2D*, predicted functional effects, eye localization, and ocular disease affinity, only 9 variants, carried by 6 genes (*CACNG8*, *PAX2*, *RXRG*, *CCDC175*, *PDE4DIP* and *LTF*), survived the filtering. These genes encode key proteins involved in cone development and survival, and retina neurotransmission. Among analyzed variants, *CACNG8*c.*6819A>T and the new *CCDC175* c.76C>T showed extremely low frequency in the control group, suggesting a key role on disease phenotypes. Such discovery could enforce the role of modifier genes into CORD onset/progression, contributing to improve diagnostic test towards a better personalized medicine.

## Introduction

Cone-rod dystrophies (CORDs) are one of the most common and heterogeneous inherited ocular diseases (IRDs) characterized by progressive retinal degeneration [1]. CORD estimated incidence ranges from 1 in 20,000–100,000, with a prevalence of 1/40.000 in Europe. It also represents one of the most common cause of legal blindness in school-aged [2]. No data is available on CORD frequency in Sicily. Degeneration could involve both eyes and it is characterized by the primary loss of cone photoreceptors, mainly due to phototransduction cascade impairments, often followed by rod involvement. CORD patients usually manifest the loss of central vision, photophobia and color vision alterations. Furthermore, due to frequent rod degeneration, early nyctalopia could be also showed by affected individuals [1].

More than 30 genes have already been associated with CORDs. These genes are mainly involved in phototransduction and related biochemical pathways, such as outer segment (OS) morphogenesis, intraflagellar transport and neurotransmitter release. Most of their causative mutations are inherited by an autosomal recessive pattern, while dominant forms are mainly related to mutations in *GUCY2D*, *PRPH2*, *CRX*, *GUCA1A*, *PROM1* genes and X-linked ones to mutations in *RPGR*, *CACNA1F*, *OPN1LW* and *OPN1MW* genes [3]. However, many other CORD causative/associated genes are still unidentified. Moreover, although the functions of some of the already known genes have been studied, it is still difficult to determine a precise genotype-phenotype correlation in CORDs, because the most of genes are associated to uncertain and/or not unique phenotypes. The existence of multigenic inheritance patterns and modifier genes makes molecular diagnoses difficult, because of phenotypic variability due to complex genetic architectures underpinning the various forms of CORDs. Locus and allelic heterogeneities limit the effectiveness of diagnostic targeted strategies, as many pathogenic variants go undetected [4]. Hence, sequencing all known genes implicated in CORDs simultaneously seems the best current approach for their definitive molecular diagnosis, and next-generation sequencing (NGS) is currently considered a powerful tool for mutation screening [5]. With about 30–60% of previously unrecognized CORD cases already characterized [6], this high-throughput method represents a promising tool for detecting novel disease-causing genes and genotype-phenotype correlation, which may lead to substantial improvement of our understanding of allele pathogenicity, protein function, and population genetics.

Here we describe a Sicilian family came to our attention as a suspected case of CORD. All affected individuals manifested different symptoms related to ocular impairment. Thus, the proband and her two sons and one daughter were screened using whole exome sequencing (WES) and whole genome sequencing (WGS), leading to the identification of guanylate cyclase 2D (*GUCY2D)* as causative gene, and of 6 novel candidate modifier genes carrying 9 previously undescribed variants.

## Materials and methods

### Family clinical data and control group recruitment

**Fig 1:**
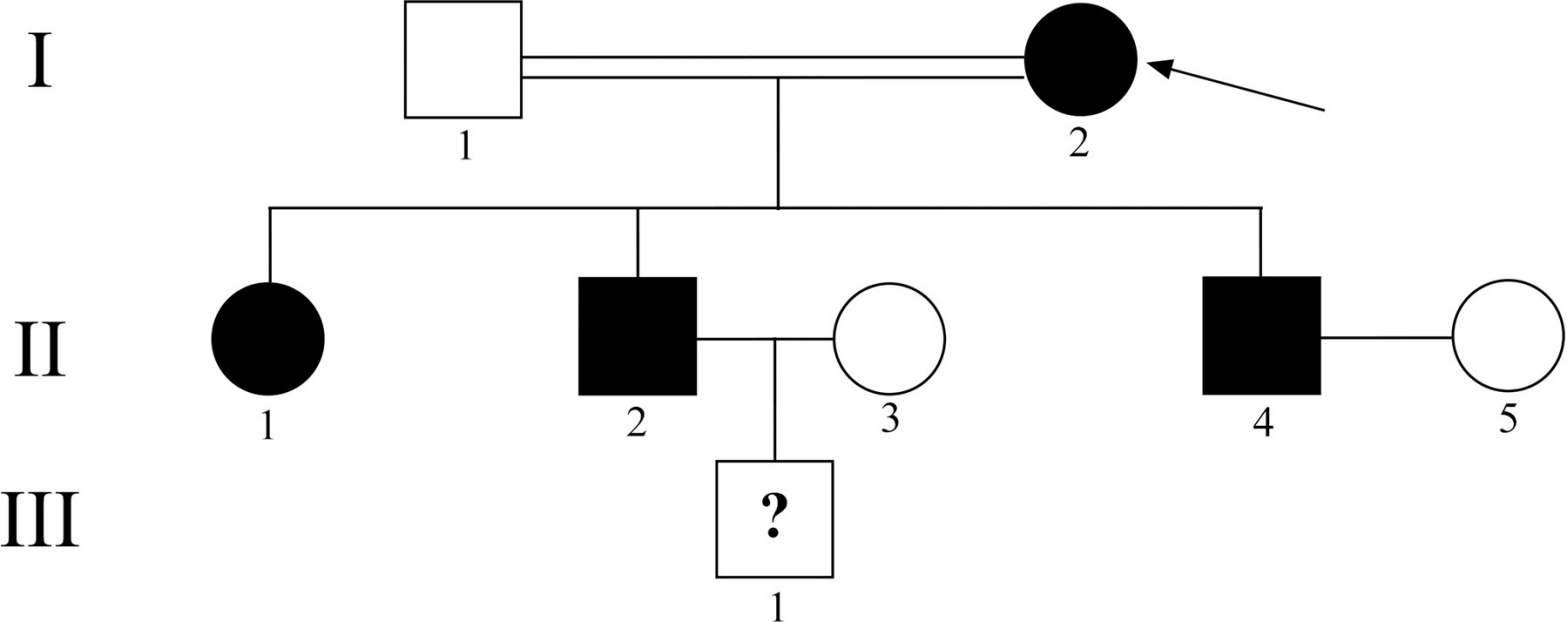
Pedigree of the Sicilian family affected by an undefined form of CORD. The affected (black fill) and unaffected (no fill) members are shown. Arrow: proband; circle: female; square: male; double line: blood relatives; question mark: uncertain phenotype.

A Sicilian family from Messina with not well identified ocular pathology, made of four affected and four unaffected individuals, was examined. Ophthalmological tests included dark adaptation and color vision, visual acuity testing (Snellen chart), and computerized visual field measurement. International Society for Clinical Electrophysiology of Vision (ISCEV) electroretinography (ERG), pattern electroretinogram (PERG), optical coherence tomography (OCT), visually evoked potential (EP), and fundus autofluorescence (FAF) were performed on four affected individuals (I2, II1, II2 and II4, Fig 1).

ERGs were recorded using DTL corneal electrodes. Amplifier bandpass settings were 0.30 to 500 Hz, and the sampling frequency was 2 kHz. Following adaptation to a uniform achromatic field (30 cd/m2), light-adapted responses were elicited by light-emitting diode (LED)-generated achromatic 3.0 cd·s/m2 flashes (4 ms) and flicker (31.25 Hz) stimuli. Subjects were, then, dark-adapted for 20 minutes, and responses were obtained by LED-generated achromatic flashes (4 ms) that ranged from 0.0001 to 10.0 cd·s/m2. All stimuli were produced by and presented in a Color-Dome ganzfeld and responses were acquired with an Espion E3 electrophysiology system (Diagnosys LLC, Lowell, MA). Reference values of ERGs were represented by an a-wave of 140–280 uV with implicit times of 15–22 ms, and by a b-wave of 350–700 uV with implicit times of 25–50 ms. For each stimulus luminance, a minimum of three responses were obtained and averaged for analysis.

The 3D SD-OCT imaging was performed by the RS-3000 Advance system (Nidek, Aichi, Japan). The OCT equipment had a 7-μm depth resolution in tissue and 20-μm transverse resolution. Each A- scan had a depth of 2.1 mm and comprised 512 pixels, providing a digital depth sampling of 4.1 μm per pixel. For wide-area 3D imaging in the posterior pole, raster scanning over a 6×6-mm square area centered on the foveal center was conducted with a scan density of 512 A-scans (horizontal) × 128 B-scans (vertical). All OCT images consisted of retinal thickness and tomographic mapping.

Clinical history of the four affected individuals was also obtained focusing on: subjective degree of vision loss, age at onset, evolution, medication, and other IRD specific clinical manifestations (such as night vision impairment, loss of peripheral vision and color perception).

A total of 100 unrelated volunteers were recruited from the same geographic area as that of the family studied and served as control group. These volunteers were subjected to the same ophthalmological tests performed on analyzed family (ERG, PERG, OCT, VEP and FAF), showing normal values for each clinical exam without any other relevant diseases. Blood samples were collected from all nine family members and all volunteers for genomic DNA sequence analysis. The research followed the tenets of the Declaration of Helsinki and was approved by the Scientific Ethics Committee of the Azienda Ospedaliera Universitaria–Policlinico “G. Martino” Messina. Informed consent was signed by all family members and controls after explanation of the nature and possible consequences of the study.

### Ion proton (WGS) and Illumina (WES) sequencing

Genomic DNA was extracted from peripheral blood using QIAamp^®^ DNA Mini Kit (Qiagen) then quantified by NanoPhotometer P-330 (Implen). DNA integrity was evaluated by visual inspection on a 1% agarose gel.

Single-end libraries for WGS were generated after fragmentation by the Ion Xpress™ Plus Fragment Library Kit (Thermo Fisher Scientific, Waltham, MA, USA) and clonal amplification by Ion PI™ Template OT2 200 Kit v3 at Ion OneTouch™ 2 Instrument (Thermo Fisher Scientific, Waltham, MA, USA). Then, three Ion PI Chip v.2 were run on an Ion Proton™ System (Thermo Fisher Scientific, Waltham, MA, USA), using the Ion PI Sequencing 200 Kit v2.

Paired-end libraries for WES were generated by the SureSelect XT HS Reagent Kit (V6) (Agilent, Santa Clara, CA, USA) kit and sequenced on a NovaSeq 6000 Illumina platform.

### Read mapping

Produced raw data were quality checked by the FastQC (v.0.11.7) tool (http://www.bioinformatics.babraham.ac.uk/projects/fastqc) and filtered according to Phred score value (reads with Phred score < 30 were trimmed, along with adaptor sequences).

Generated sequences were processed in-house using the bioinformatics software CLC Genomics Workbench 22.0.0 [7]. The analytic pipeline foresaw the alignment to the GRCh38 Human Reference Genome (<3 mismatches/100 bp per alignment), followed by duplicate read removal, InDel realignment and Base Recalibration, before variant calling. Variant calling using the fixed ploidy variant detection was performed, reporting variants with >90% probability. Pyro-error variants were removed through specific filtering (parameters set: “in homopolymer regions with minimum length = 3”; “with frequency below = 0.8”). The algorithm behind the Fixed Ploidy Variant Caller combines a Bayesian model (examining posterior probabilities) with a maximum likelihood approach.

Finally, found variants were annotated by ANNOVAR v.20220330 tool and included databases [8].

### Filtering candidate variants

Firstly, variants from both exome and genome sequencing of proband were matched and merged. The first criterion we used to select candidate variants was to determine if newly identified variants had already been associated to CORD or other Inherited Retinal Diseases (IRDs) and their frequency, thanks to explored reference databases included in ANNOVAR (ClinVar, dbSNP, 1000 genomes, HapMap, dbnsfp30a), along with HGMD Professional [9] and RetNet (https://sph.uth.edu/retnet/) databases.

Then, variants were analyzed to assess the potential impact on encoded gene function, evaluating: 1) Coding region variants; 2) Variants potentially involved in splice site alterations; 3) Regulative variants (3’ and 5’ UTR); 4) Variants with high conservation score. A comparison of exome-derived variants between all analyzed exomes was also realized.

### Splicing variant effect prediction

To analyze the possible effects of identified splicing variants, the Alternative Splice Site Predictor (ASSP) tool was exploited. ASSP predicts putative alternative exon isoform, cryptic, and constitutive splice sites of coding exons. Coding sequences are generally characterized by trinucleotide frequencies which differ markedly from the frequencies observed in non-coding sequences. Codon usage reflects the probability of a sequence to be coding by comparing the observed trinucleotide frequencies with those observed in coding sequences. Codon usage values above zero indicate, that a subsequence is probably coding, while sequence showing values below zero are probably not coding (ASSP calculates log-likelihood values). Codon usage is calculated for a sliding window of a given size. Codon usage and stop codons are calculated for all three possible reading frames (F1—frame 1, F2—frame 2, F3—frame 3). Stop codons should usually not be observed within exons read in the corresponding frame. Splice sites, especially constitutive ones, are usually detected near the putative boundaries of exons, as being indicated by high codon usage values.

### Sanger validation and healthypopulation screening

Variants filtered in family affected members were validated by Sanger sequencing. Primers were designed by Primer3 tool [10], following standard settings and, then, carefully manually checked.

Amplification of gene fragments with selected variants was performed using primer pairs designed according to corresponding nucleotide sequences in GenBank. The Polymerase Chain Reaction (PCR) mix was prepared adding 1μg of genomic DNA to 50μl reaction mixture containing a 0.2 μm concentration of each primer and 1 U ACCUZYME™ DNA Polymerase (Bioline). After an initial denaturation step at 95˚C for 1 min, the samples were subjected to 35 cycles of amplification consisting of 15 sec of denaturation at 95˚C, 10 sec of annealing. The annealing temperature was optimized for each primer set. Following PCR, 5 μl of amplified product was examined by electrophoresis on a 1% agarose gel. Sanger sequencing was, then, performed using the BigDye Terminator© v3.1 Cycle Sequencing Kit chemistry and run on a 3130xl Genetic Analyzer (Applied Biosystems, Thermo Fisher Scientific). Primer list and PCR thermal conditions are available upon request.

Then, confirmed variants were searched in healthy family individuals and in a group of 100 healthy subjects from Messina, in order to validate the analyzed variants as disease related.

### Cluster analysis by pathway functional enrichment

To determine if different observed clinical phenotypes could be influenced by the combined effects of more than one variant in modifier genes, a cluster analysis by pathway enrichment was realized. This was carried out by Cytoscape (v3.8.2) [11] and its plugins GeneMANIA (v3.5.2) [12], ClueGO (v2.5.7) and CluePedia (v1.5.7) [13]. Subcellular locations, biological processes, molecular function, and KEGG pathways, which were inferred from electronic annotation and experimental data, were all in the identified GO categories. A minimum level of 3 and a maximum level of 8 were set as the GO level interval with a minimum of two genes per category. Our experimental data were directly compared and enriched with publicly available information from STRING [14], IntAct [15], MiMI [16], miRBase [17], and miRecords [18]. miRNAs target site analysis was performed using the PolymiRTS Database (v3.0) [19].

### Data mining and statistical analysis

Qualitative association analysis on the 32 selected variants was performed by means of MatLab software package (R2021a, The MathWorks Inc., Natick, MA, 2000); for statistical analysis we made use of IBM SPSS software package (IBM Corp. Release 2016. IBM SPSS Statistics for Macintosh, Version 26.0. Armonk, NY: IBM Corp).

To investigate the complex phenotype—genotype patterns, and due to the relatively low sample size investigated, we adopted a qualitative approach: for each genotype *v*, we clustered patients showing a given symptom *s* from those who did not. By considering an additive model (1 = wild-type, 2 = heterozygous, 3 = homozygous mutated), which implies that the risk conferred by an allele is increased 1r-fold for heterozygotes and 2r-fold for homozygotes, we extracted and averaged genotype from each group and calculated the difference d_v_^s^. A positive sign indicates that affected patients showed a genotype more directed towards homozygous mutation if compared to unaffected patients; vice versa, a negative sign indicates that unaffected patients showed a genotype preferentially directed towards heterozygous mutation. Positive values may therefore appear in variants involved in symptom manifestation, whereas negative differences may highlight variants whose presence may have a protective role in symptom arousal.

A weight p_v_^s^ was subsequently created to better define the magnitude of the difference d_v_^s^:p_v_^s^ was assigned a value of 0 when abs(d_v_^s^) < 0.5, alternatively a value of 1 was considered when 0.5 ≤ abs (d_v_^s^) < 1. If abs ≥ 1, p_v_^s^ was set to 2. By gathering the weights obtained from each genotype v and symptom s, we therefore obtained a matrix vector P_s,v_ = [p_v_^s^], with row elements s representing symptom s, while columns v represent scores for genotypes. The usefulness of the matrix consists in the fact that by summing over the v-th column we are able to obtain a score SC^v^_pol_ whose magnitude is proportional to its relevance in clustering both affected and unaffected patients given the whole patterns of symptoms.

## Results

### Clinical analysis of family affected members evidenced a very complex pattern of pathological phenotypes

The Sicilian family showed a dominant inheritance pattern, with a possible incomplete penetrance in the last generation (Fig 1), where the III1 subject, who is apparently healthy, could be too young to develop symptomatology.

The most of clinical features of the four affected family members were typical of those associated to cone-rod dystrophy, such as night blindness from birth, photophobia, generalized dyschromatopsia and progressive loss of visual field and visual acuity, undetectable ERG and macula with prominent atrophic macular lesion (also called "macular colobomas"), even if many aspects remain unclear and different throughout the family patients (Table 1).

**Table 1:** Clinical features of the four affected members of the reported Sicilian family.

SIGN, SYMPTOMS, OTHER FEATURES	PATIENT			
	I2	II1	II2	II4
AGE AT EXAMINATION	66	42	35	21
NIGHT BLINDNESS	Yes	No	No	Yes
NARROWING OF VISUAL FIELD	No	No	No	No
PHOTOPHOBIA	Yes	Yes	Yes	Yes
BEST CONDITION OF VISUAL ACUITY	Penumbra (Darkness until 50 years)	Penumbra	Darkness	Penumbra
VISION ACUITY	1/20	2/20	4/20	2/20
AGE OF ONSET	First year of life	First year of life	First year of life	First year of life
PATTERNS OF VISUAL FIELD LOSS	Cecocentral scotoma	Cecocentral scotoma	Cecocentral scotoma	Cecocentral scotoma
GREEN->LIGHT BLUE (Altered colour perception)	Yes	Yes	No	No
BLACK->BLUE (Altered colour perception)	No	Yes	No	Yes
WHITE PERCEPTION (Altered colour perception)	No	Yes	No	No
RED->ORANGE (Altered colour perception)	No	No	Yes	Yes
YELLOW (BY COMPARISON) (Altered colour perception)	No	Yes	Yes	No
OPTICAL NERVE ATROPHY	No	Yes (marked ischemia)	No	Yes (subatrophied and pale)
BILATERAL AND HORIZONTAL NYSTAGMUS	Yes	Yes	Yes	Yes
MACULAR DEGENERATION	Macular and perimacular	Macular	Macular	Macular
HEAD MOVEMENT (TO IMPROVE FOCUS)	Yes	No	Yes	No
PHOTOPSIA	Yes	Yes	No	No
SYMPTOMATOLOGY PROGRESSION	Up to 15 years walking well in the dark; gradual reduction of vision, up to only light perception by 45 years; today blindness in the dark	Normal vision up to high school, then lack of focus; at university did not read any more, high photophobia; today more difficulty in vision, much more photophobia, blurred vision	Gradually worsening of vision until present day	From 12 years onwards significant photophobia and following worsening of vision
CURRENT THERAPY	Antioxidants	/	/	Antioxidant (astaxanthin)
OTHER OCULAR DISEASES	Bilateral cataracts	Astigmatism (+1.50), Myopia (-5.00)	/	Astigmatism (+1.50), Myopia (-6.00)
OTHER DISEASES	Bilateral hearing loss, Hypertension, Diabetes mellitus type 2, Allergies, Appendectomy	Multinodular goiter, Chronic otitis, adrenocortical hypertrichosis, Appendectomy	/	Appendectomy

Signs, symptoms, and clinico-pathological features listed are those most frequently observed in CORDs, as well as those specific of reported cases. Best condition of visual acuity refers to slit-lamp exam. Vision acuity is measured as Snellen equivalents. Dark Adaptation refers to scotopic ERGs normal values. Visual field was measured by Goldmann Visual Field Test. Color perception evaluation was performed using Ishihara plates. Optical nerve compromission was diagnosed by ophthalmoscopic exam and diffusion-weighted MRI. Astigmatism and myopia were measured in diopters.“/” = absence of feature. Patients’ IDs refer to Fig 1.

**Fig 2:**
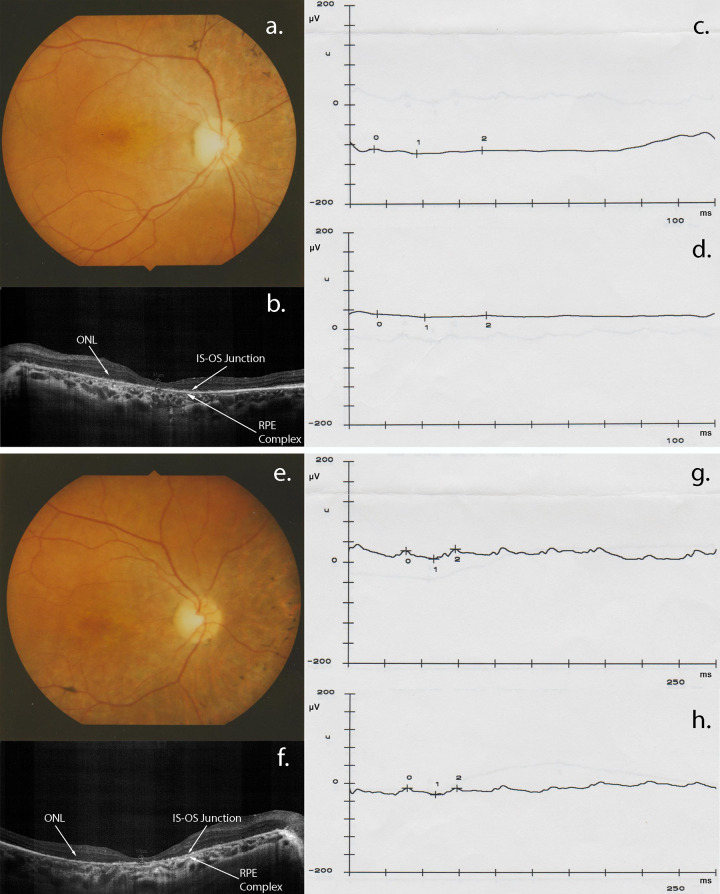
Proband’s fundus, optical coherence tomography (OCT) and electroretinogram (ERG). Fundus photo of left eye is shown upside down. Fundus examination demonstrated mild changes typical of RP in the left (a) and the right (e) eyes, including slightly pale optic discs with almost normal retinal vessels, generalized pigmentary granularity, and moderate bone-like spicules in peripheral areas. The OCT scans of the left eye (b) and the right eye (f) revealed decreased macular thickness, as well as a severe loss of EZ line and outer segments. Scotopic (c, g) and photopic (d, h) ERGs revealed a generalized cone dysfunction with rod involvement. ONL = Outer Nuclear Layer. IS-OS Junction = Inner Segment-Outer Segment Junction. RPE Complex = Retinal Pigment Epithelium Complex.

The proband became completely blind at 50 years of age, and fundus examination showed tapetal retinal degeneration, macular pigmentary dystrophy, pale disc and retinal pigmented epithelium atrophy (Fig 2). OCT showed a relevant ellipsoid band loss in outer retina, choroid thickness and a thinning of retinal pigment epithelium (RPE) in both eyes. Additionally, ERGs revealed a generalized cone dysfunction with rod involvement (photopic and scotopic extinct ERGs), with compromised visual response (the tracings showed no response). Clinical examination results of the other affected member are shown in S1–S3 Figs. The remaining four unaffected individuals, instead, showed normal phenotype in all ophthalmological examinations.

### NGS data analyses identified possible CORD causative and modifiers genes

**Fig 3:**
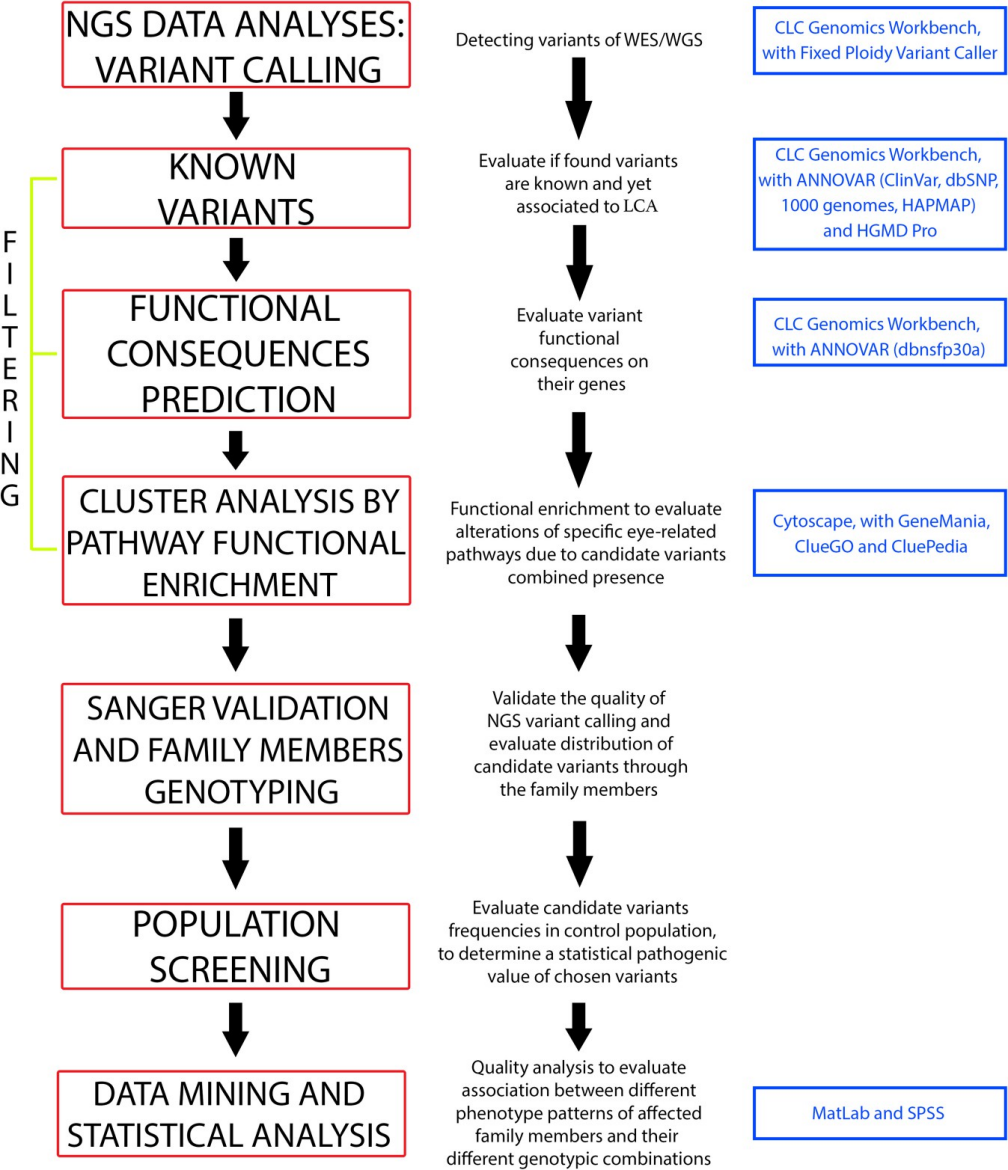
Graphical workflow of data analyses. Data analyses involved several filtering levels, to obtain final candidate variants.

WES was initially performed on the proband, then on her 2 sons and 1 daughter. A mean value of 92,806,962 paired-end reads (mean length ~ 150 bp) were produced, considering all exomes. About 92.14% of these reads overcame the average quality score (Phred score) of 30, permitting to detect a mean of 43,921 variants, of which 38,678 single nucleotide variants (SNVs) and 3,941 insertions or deletions (InDels). Subsequently, a total of 172,372,728 single end reads (mean read length ~ 150 bp) were generated for proband’s whole genome. This time, an inferior percentage of reads (~ 73%) were characterized by a Phred score ≥ 30, permitting us to identify a total of 20,306 variants, including 17,501 SNVs and 2,805 InDels. About the 80% of coding sequence variants found in WGS was also detected in proband’s exome. A graphical workflow of the whole down-stream data analysis is represented in Fig 3.

**Fig 4:**
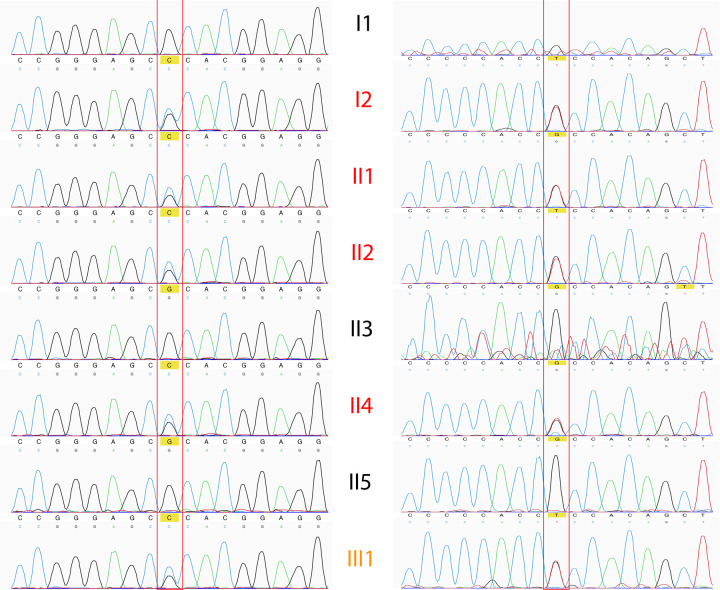
Sanger validation of candidate *GUCY2D* causative variants. Both *GUCY2D* identified variants (c.2513G>C on the left, and c.3044-7G>T on the right) were validated by Sanger sequencing in all family members. Black text = Healthy family member; Red text = Affected family member. Orange text = Uncertain phenotype in this family member.

**Fig 5:**
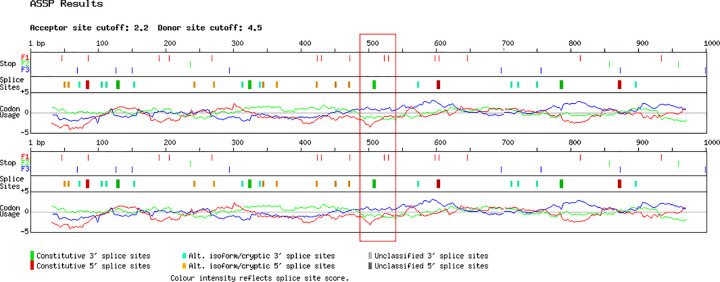
Alternative splicing predictive analysis for *GUCY2D* c.3044-7G>T. The red box highlights the differences between codon usage of *GUCY2D* wild-type (at the top) versus *GUCY2D* carrying the splice variant c.3044-7G>T (at the bottom), suggesting the possible alternative splicing event of mutated gene. More details through the text.

Using RetNet and Human Genetic Mutation Database (HGMD) Professional, and analyzing the distribution through the affected family members, we found that the causative variant probably responsible of the CORD onset could be the variant c.2513G>C in *GUCY2D* gene. The c.3044-7G>T variant, also carried by *GUCY2D* and detected in all affected patients, might probably exert a modifier role (_((((xxx))))_)[20–36] (Fig 4). Using ASSP, the splice variant c.3044-7G>T, present within the sequence of the constitutive acceptor splice site “acctccacagCTTACCGCAT”, seems to increase the 40% possibility to perform an alternative splicing event for *GUCY2D* (Fig 5, S1 Table). No other variants in known CORD causative genes were found.

However, in order to explain the different phenotypes exhibited by affected members of analyzed family, we focused on other genes which could act as modifiers ones, affecting the phenotypic and/or molecular expression of previously cited genes.

According to a preliminary filtering based on relationship with *GUCY2D*, functional effects, eye localization and ocular disease affinity, 32 candidate variants, distributed within 10 candidate modifier genes, were classified as new missense and UTR mutations (Group 1); missense, nonsense, and inframe SNPs (Group 2); splice-site and UTR regulation site SNPs (Group 3); and synonymous SNPs (Group 4). Detailed features of such variants and their distribution throughout the family are shown in Table 2.

**Table 2:** Preliminary candidate variants genotyping and distribution through the members of the reported family.

Source	Gene	SNP ID	Variant Conseq.	HGVS	Protein Mutation	HGMD Pathog.	gnomAD AF	Clinvar	Phenotype	*AFFECTED*				*HEALTHY*			*UN*.
										I2	II1	II2	II4	I1	II3	II5	III1
**CAUSATIVE**	***GUCY2D***	rs61750173	Missense	c.2513G>C	Arg838Pro	DM	/	P/LP	CORD	**+/-**	**+/-**	**+/-**	**+/-**	**+/+**	**+/+**	**+/+**	**+/-**
**ASSOC.**		rs56348143	Splice Region	c.3044-7G>T	/	/	0.04030	B	/	**+/-**	**+/-**	**+/-**	**+/-**	**+/+**	**+/+**	**+/+**	**+/-**
**CANDIDATE VARIANTS IN MODIFIER GENES**	*ABCA4*	rs1800717	Splice Region	c.6730-3T>C	/	/	0.1421	B	CORD, MD, STGD	**+/-**	**+/-**	**+/-**	**+/-**	**+/+**	**+/+**	**+/+**	**+/+**
	*PCDH15*	rs4935502	Missense	c.1304A>C	Asp435Ala	/	0.1759	B	USH 1F	**+/-**	**+/-**	**+/+**	**-/-**	**+/-**	**+/+**	**+/+**	**+/+**
	*RP1*	rs446227	Missense	c.5008 G>A	Ala1670Thr	/	0.2202	CONFL.	RP	**+/-**	**+/-**	**+/-**	**-/-**	**-/-**	**+/+**	**+/-**	**+/+**
		rs444772	Missense	c.2615G>A	Arg872His	/	0.2497	B	RP	**+/-**	**+/-**	**+/-**	**-/-**	**-/-**	**+/-**	**+/-**	**+/+**
		rs414352	Missense	c.5071T>C	Ser1691Pro	/	0.2573	B	RP	**+/-**	**+/-**	**+/-**	**-/-**	**-/-**	**+/+**	**+/-**	**+/+**
		rs441800	Synonymous	c.5175A>G	Gln1725 =	/	0.2141	B	RP	**+/-**	**+/-**	**+/-**	**-/-**	**-/-**	**+/+**	**+/-**	**+/+**
		rs61739567	Missense	c.6098G>A	Cys2033Tyr	/	0.3025	B/LB	RP	**+/-**	**+/-**	**+/-**	**+/+**	**+/+**	**+/-**	**+/-**	**-/-**
		rs10654889	3’ UTR, Regulatory	c.*261dupT	/	/	0.1111	LB	RP, CORD	**+/-**	**+/-**	**+/-**	**+/-**	**+/-**	**+/-**	**+/-**	**+/-**
	*PAX2*	rs7094977	5’ UTR, Regulatory	c.-375C>A	/	/	0.7981	/	/	**+/-**	**+/+**	**-/-**	**+/-**	**-/-**	**-/-**	**-/-**	**-/-**
	*RXRG*	rs283696	Splice Region	c.875+6A>G	/	/	0.8235	/	/	**+/-**	**-/-**	**+/-**	**-/-**	**-/-**	**-/-**	**-/-**	**+/-**
	*CACNG8*	rs66507429	3’ UTR, Regulatory	c.*6819A>T	/	/	0.1672	/	/	**-/-**	**-/-**	**-/-**	**-/-**	**+/+**	**+/+**	**+/+**	**+/+**
		rs4806479	3’ UTR, Regulatory	c.*6802G>A	/	/	0.3276	/	/	**+/+**	**+/+**	**+/+**	**+/+**	**-/-**	**+/+**	**-/-**	**+/+**
		rs4806480	3’ UTR, Regulatory	c.*6870C>T	/	/	0.3709	/	/	**+/+**	**+/+**	**+/+**	**+/+**	**-/-**	**+/+**	**+/+**	**+/+**
	*CC2D2A*	rs13121363	Splice Region	c.3183-8T>C	/	/	0.6913	B/LB	JBTS, MKS	**+/-**	**+/-**	**+/-**	**-/-**	**-/-**	**+/+**	**+/-**	**+/-**
		rs73125627	Synonymous	c.3201G>A	Ser1067 =	/	0.2029	B	JBTS, MKS	**+/-**	**+/-**	**+/-**	**+/+**	**+/+**	**+/-**	**+/-**	**+/+**
	*CCDC175*	rs7141565	Regulatory	c.-79C>G	/	/	0.2894	/	/	**+/+**	**+/-**	**+/-**	**+/-**	**-/-**	**-/-**	**+/+**	**-/-**
		rs4898996	Synonymous, Regulatory	c.72T>C	Thr24 =	/	0.9163	/	/	**-/-**	**-/-**	**-/-**	**-/-**	**-/-**	**-/-**	**-/-**	**-/-**
		NEW	Missense	c.76 C>T	Pro26Ser	/	/	/	/	**+/-**	**+/+**	**+/-**	**+/-**	**+/+**	**+/+**	**+/+**	**+/+**
	*PDE4DIP*	NEW	Missense	c.6967 G>A	Asp2323Asn	/	/	/	/	**+/-**	**+/-**	**+/-**	**+/-**	**+/-**	**+/-**	**+/-**	**+/-**
		rs587647089	Missense	c.7015C>A	Arg2339Ser	/	0.00005257	/	/	**+/-**	**+/-**	**+/-**	**+/-**	**+/-**	**+/-**	**+/-**	**+/-**
		NEW	Missense	c.7025 A>G	Gln2342Arg	/	/	/	/	**+/-**	**+/-**	**+/-**	**+/-**	**+/-**	**+/-**	**+/-**	**+/-**
		rs1055312	Stop Gained	c.7052G>A	Trp2351Ter	/	0.0001051	/	/	**+/-**	**+/-**	**+/-**	**+/-**	**+/-**	**+/-**	**+/-**	**+/-**
		NEW	3’ UTR	c.*33 G>T	/	/	/	/	/	**+/-**	**+/-**	**+/-**	**+/-**	**+/-**	**+/-**	**+/-**	**+/-**
		rs141564171	3’ UTR	c.*39G>A	/	/	0.08689	/	/	**+/-**	**+/+**	**+/+**	**+/+**	**+/+**	**+/+**	**+/+**	**+/+**
		rs372847794	3’ UTR	c.*60A>T	/	/	0.003043	/	/	**+/-**	**+/-**	**+/-**	**+/-**	**+/-**	**+/-**	**+/-**	**+/-**
		rs587638515	3’ UTR	c.*80G>C	/	/	0.0001643	/	/	**+/-**	**+/-**	**+/-**	**+/-**	**+/-**	**+/-**	**+/-**	**+/-**
		NEW	3’ UTR	c.*105G>A	/	/	/	/	/	**+/-**	**-/-**	**+/-**	**+/-**	**-/-**	**+/-**	**-/-**	**+/-**
		NEW	3’ UTR	c.*184 C>A	/	/	/	/	/	**+/-**	**+/-**	**+/-**	**+/-**	**+/-**	**+/-**	**+/-**	**+/-**
	*LTF*	rs10662431	Inframe Insertion	c.68_69insAAG	/	/	0.9815	/	/	**-/-**	**-/-**	**-/-**	**-/-**	**-/-**	**-/-**	**-/-**	**-/-**
		rs777783348	Synonymous	c.84C>T	Cys28 =	/	0.00002296	/	/	**+/-**	**+/-**	**+/+**	**+/-**	**+/+**	**+/+**	**+/+**	**+/+**
		rs1126477	Missense	c.85G>A	Ala29Thr	/	0.5295	/	BLP	**+/-**	**+/+**	**+/-**	**+/+**	**+/-**	**+/-**	**+/+**	**+/-**
		rs1126478	Missense	c.140A>G	Lys47Arg	/	0.5082	/	BLP	**+/-**	**+/+**	**+/-**	**+/+**	**+/-**	**+/-**	**+/+**	**+/-**

The probably causative variant is carried by GUCY2D (c.2513G>C), which also presents the probably associat-edc.3044-7G>T splicing variant (indicated as “ASSOC.”). The other 32candidate variants are carried by ten putative modifiers genes emerged from NGS analyses. “UN.” = Uncertain phenotype (healthy at this moment). “/” = absence of feature. “DM” = Disease Mutation (HGMD). “DM?” = Disease Mutation without certain evidence. “P = Pathogenic”. “LP = Likely pathogen-ic”. “B = Benign”. “LB = Likely benign”. “CONFL. = Conflicting interpretation of pathogenicity”. “CORD = Cone-rod dys-trophy”. “MD = Macular degeneration”. “RP = Retinitis pigmentosa”. “STGD = Stargardt disease”. “USH = Usher syndrome”. “JBTS = Joubert syndrome”. “MKS = Meckel-Gruber syndrome”. “BLP = Blood protein levels association”. “+/+” = wild type. “+/-”= heterozygous. “-/-”= mutated homozygous.

### Pathway functional enrichment permitted to filter out nine final candidate variants carried by six potential modifiers genes

**Fig 6:**
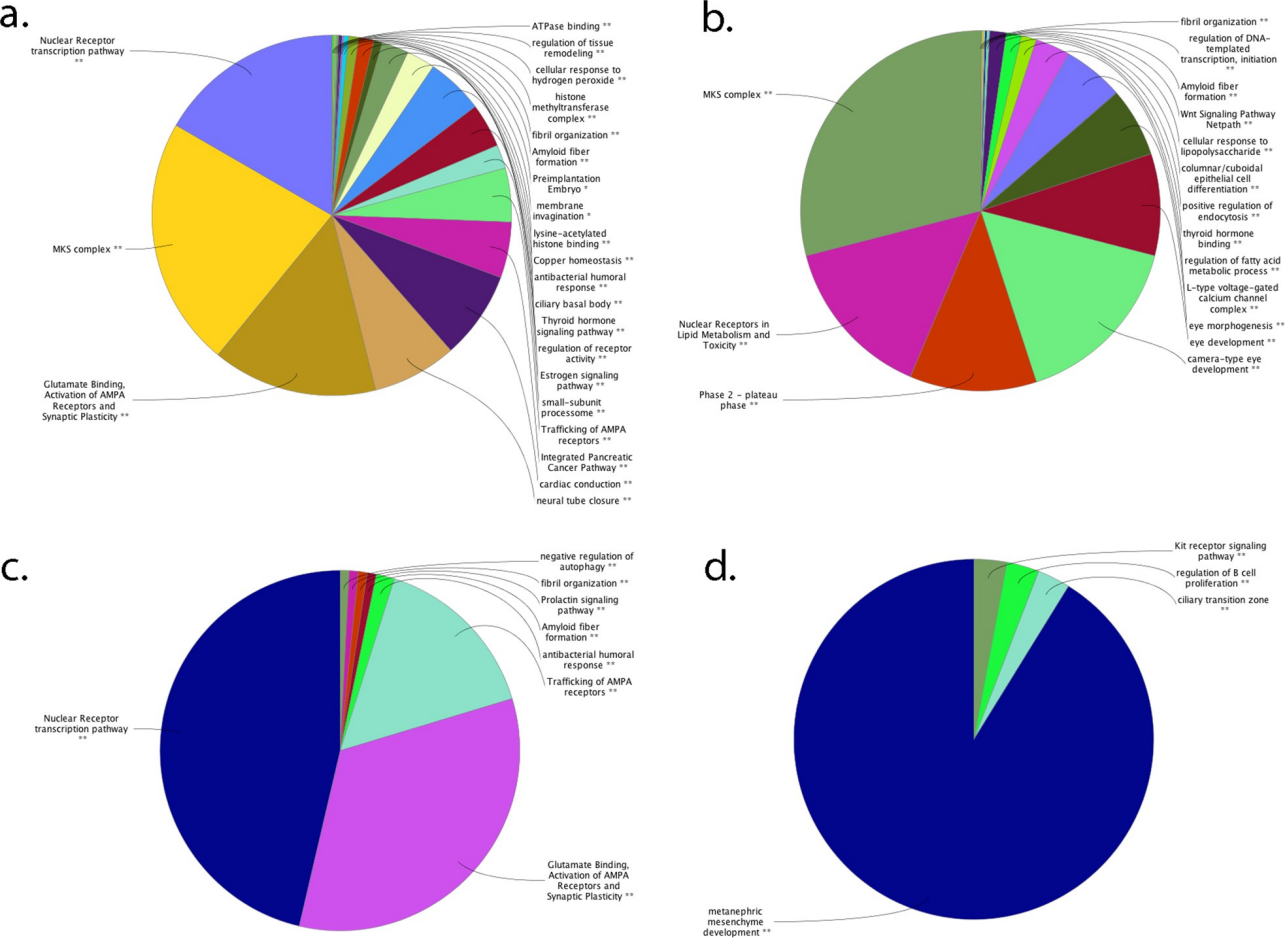
Functional groups in ClueGO overview. The pie charts present functional groups for analyzed genes, based on GO, KEGG, Reactome and WikiPathways annotation by ClueGO Cytoscape plug-in, then clustered into STRING action enriched (a), combined score (b), database score (c), and textmining (d) categories. The name of the group is given by the group leading term (the most significant term in the group). The group sections correlate with the number of the terms included in the group. Network structure of functional groups is calculated on kappa Score, which shows the relationships between the terms based on their overlapping genes.

The cluster analysis by pathway functional enrichment conducted by Cytoscape and its GeneMANIA plug-in revealed that relevant genetic and physical interactions connect almost the entire set of analyzed genes, suggesting a common involvement in multiple pathways (S4 Fig). Then, basing on eye and vision relationship computed by ClueGO, identified pathways were subcategorized into 87 main hierarchically structured GO classifications, including 59 biological processes, 13 cellular components, 4 immune system processes, and 11 molecular functions (Fig 6).

Two KEGG pathways, 12 Reactome pathways, and 2 WikiPathways emerged from analysis (S2 Table). The overall results indicated that the identified genes belonging to these GO categories may play the most important roles in regulation of eye/retina development, homeostasis and functionality, especially in synaptic activities and ciliary transport.

Furthermore, the CluePedia enrichment analysis highlighted ninety-three terms connected by 247 edges (activation, binding, catalysis, expression, inhibition, ptmod, reaction) with the kappa scores, and showed relevant enrichment (p < 0.05) in the identified protein interactome (S5 Fig).

By CluePedia Cerebral plug-in layout [37] it was also possible to establish that most of the protein encoded by the 8 genes are located in intracellular compartments, with only one (CCDC175) that could be extracellular and one (CACNG8) that could act at the plasma membrane level (S6 Fig). By using the mIRANDA database we showed that from 2 to 9 miRNAs could regulate gene expression of each analyzed gene (S7 Fig). These data are supported by the PolymiRTS Database, which indicates that the presence of *CACNG8* c.*6819A>T and *PDE4DIP* c.*39G>A could create or disrupt several miRNAs target sites (Table 3).

**Table 3:** PolymiRTS analysis of miRNAs potentially altered target sites associated with the reported candidate variants.

Gene	Location	dbSNP ID	Variant type	Wobble Base Pair	Ancestral Allele	Allele	miR ID	Conservation	miRSite	Functional Class	Experimental Support	Context+ Score Change
*CACNG8*	54492922	rs66507429	SNP (A>T)	N	A	A	hsa- miR- 6893- 3p	5	aaacgC***A***GCAGGc	D	N	-0.036
*PDE4DIP*	144852315	rs141564171	SNP (G>A)	Y	G	G	hsa- miR- 378a- 3p	6	gggagg***G***TCCAGA	D	N	-0.124
							hsa- miR- 378b- 3p	6	gggagg***G***TCCAGA	D	N	-0.121
							hsa- miR- 378c- 3p	6	gggagg***G***TCCAGA	D	N	-0.124
							hsa- miR- 378d- 3p	6	gggagg***G***TCCAGA	D	N	-0.124
							hsa- miR- 378e- 3p	6	gggagg***G***TCCAGA	D	N	-0.124
							hsa- miR- 378f- 3p	6	gggagg***G***TCCAGA	D	N	-0.124
							hsa- miR- 378h- 3p	6	gggagg***G***TCCAGA	D	N	-0.124
							hsa- miR- 378i- 3p	6	gggagg***G***TCCAGA	D	N	-0.124
							hsa- miR- 422a- 3p	6	gggagg***G***TCCAGA	D	N	-0.124
							hsa- miR- 4638- 3p	6	gggagg***G***TCCAGA	D	N	-0.12
							hsa- miR- 711	6	gggaGG***G***TCCAga	D	N	-0.178
						A	hsa- miR- 378j- 3p	6	gggagg***A*** TCCAGA	C	N	-0.085
							hsa- miR- 4782- 5p	6	gggagg***A*** TCCAGA	C	N	-0.125
							hsa- miR- 5706	6	gggagg***A*** TCCAGA	C	N	-0.118
							hsa- miR- 6749- 3p	4	GGGAGG***A***tccaga	C	N	-0.187
							hsa- miR- 6839- 5p	10	gggagg***A*** TCCAGA	C	N	-0.088

Gene: Gene symbol. Location: variant location in the mRNA transcript. dbSNP ID: ID associated to dbSNP. Variant type: variant category. Wobble Base Pair: whether the variant can form a G:U wobble base pair with the miRNA. Ancestral Allele: if applicable, the ancestral allele is shown. Allele: two alleles of the variant in the mRNA transcript. miR ID: ID associated to miRBase. Conservation: conservation score of the miRNA site. miRSite: sequence context of the miRNA site. Bases complementary to the seed region are in capital letters and variants are highlighted with bold, italics, and underlined letters. Functional Class: effect of variant presence on miRNAs target sites (D: disruptive of a conserved miRNA site; C: the derived allele creates a new miRNA site). Experimental Support: highlights the experimental support to predicted miRNAs analysis (N: absent). Context+ Score Change: it predicts the binding of a miRNA to the entire 3’-UTR by summing over contributions made by individual sites within the 3’-UTR that have perfect sequence complementarity to the miRNA seed region. A more negative value of the context+ score difference indicates an increased likelihood that the miRNA targeting is disrupted or newly created by the mutation in the target sites. The presence of CACNG8 c.*6819A>T and PDE4DIP c.*39G>A could create or disrupt several miRNAs target sites.

**Fig 7:**
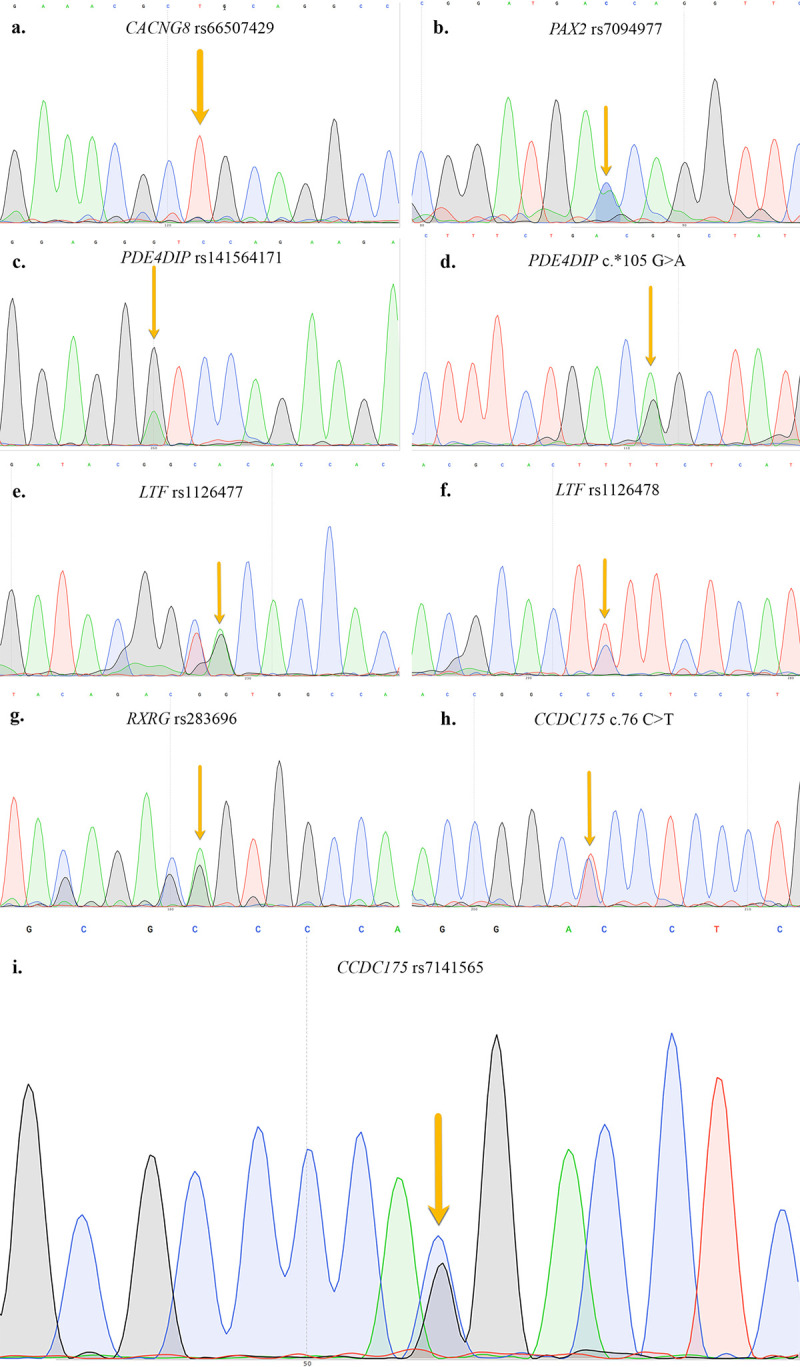
Sanger validation of final modifier gene candidate variants. All nine final selected variants carried by candidate modifier genes were validated by Sanger sequencing, and electropherograms are reported in figure. a–h, variants validated in proband; i validated in one proband’s son.

Finally, 9 variants carried by 8 candidate modifiers genes were filtered out and, soon after, validated by Sanger sequencing (Fig 7).

### Data mining and statistical analysis

**Fig 8:**
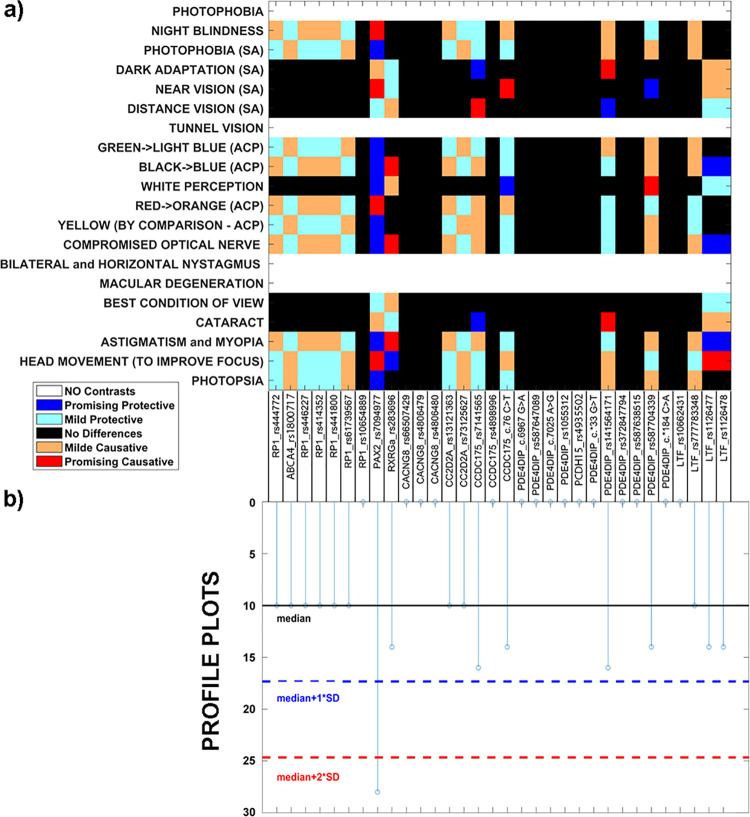
Symptom patterns and variants. a) By considering an additive model, for each symptom we calculated difference between average genotype for affected vs unaffected patients. This value was then assigned a score according to effect size (mild = 0.5, promising> = 1). Score is shown on a heatmap that reflects the strength (mild or strong) and putative causative (reds), or protective (blues) role of examined variants. White rows arise when all patients show a given symptom, thus making comparison of genotype distribution impossible. b) Stem plot shows total score obtained for a given variant when considering absolute value of effect sizes gathered across all symptoms; this measure represents the overall relevance of that variant in explaining symptom patterns. Lines highlight median (black), more one (blue) and two (red) standard deviations with respect to score distribution.

Once established that the selected genes were involved in common eye related pathways, it was necessary to determine the genotype–phenotype association with the pathology. We analyzed the 32 variant genotypes in all family members and noticed that the only variant with different distribution between healthy and sick subjects was *CACNG8* c.*6819A>T (S8 Fig). This observation was statistically confirmed by Fischer exact test, which associated the variant to disease (p-value = 0.040). However, these data did not suffice to explain the wide spectrum of different CORD phenotypes. To discriminate family phenotypes, we tried to associate other selected variants to clinical symptoms, which were quite varied in the affected family members. Fig 8 shows a comprehensive graphical overview explaining the calculations for all symptoms and variants. For each element of heat map (Fig 8A), we assigned different colors to differentiate between:

- irrelevant differences between affected and unaffected groups (-0.05≤d<0.05, black);
- low differences between groups, likely having a pathogenic, causative (0.05≤d<1, orange), or a protective (-1<d≤-0.05, cyan) role against symptom manifestation;
- relevant differences between groups, linked to stronger evidence of a pathogenic, causative (d> = 1, red) or protective (d≤-1, blue) role against symptom manifestation.

In Fig 8A, white stripes correspond to symptoms that were common to all patients; in such cases, a differentiation between affected and unaffected groups could not be obtained. In Fig 8B, we show scores assigned to each variant with a stem plot; a high score indicates that the presence of such variant likely plays a relevant role in symptom manifestation and/or absence. From Fig 8B, it is clear that some variants play a bigger role than others in symptom patterns. To help interpretation, in Fig 8B we further represented median score by means of horizontal line median score (black line), as well as the summation of median score with one (blue line) and two (red line) standard deviations. According to these analyses, it resulted that *CACNG8* c.*6819A>T departs two standard deviations from the median, while the other eight genes (*PAX2* c.-375C>A, *RXRG* c.875+6A>G, *CCDC175* c.-79C>G and c.76 C>T, *PDE4DIP* c.*39G>A and c.*105 G>A, *LTF* c.85G>A and c.140A>G) fall between one and two standard deviations from the median. No other significant differences were observed for the other variants. These results led us to the idea that the heterogeneity of the phenotypes could be determined by the above variants, differently distributed amongst the family patients. Moreover, to highlight the similarity of family components, a clustering based on all variant genotypes (S9A and S9B Fig) and symptom (S9C and S9D Fig) distribution was performed, with a cut-off of 70% and 80% respectively, which revealed that the proband and two sons (II1 and II2) cluster together genetically, as (II3 and III1), while common clinical findings are found for I1, II3, II5 and III1, who appear healthy.

### Population screening

Based on Exome Variant Server (http://evs.gs.washington.edu/EVS/), for all the variants identified, minor allele frequency (MAF) was at least 5% in all populations assayed. To determine the frequency of selected variants within the population of Messina, we performed a population screening, considering an unrelated healthy donor group (n = 100) born and living in Messina for at least two generations. In detail, especially for *CACNG8* c.*6819A>T and *CCDC175* c.76 C>T, the survey revealed a very low frequency distribution, with the latter was totally absent in unaffected people (S10 Fig).

## Discussion

CORD is a highly heterogeneous disease in clinical features and genetics, and association of mutations/variants in at least 30 genes to the various forms of the disease is not immediate [20]. Furthermore, it is highly likely that mutations/variants in other still unidentified genes, as well as non–coding RNAs [38, 39], could contribute to CORD pathogenesis. The complexity of the situation is compounded by the variety of genotype-phenotype combinations that occur. Heterogeneity depends on various factors. First, the allelic factor: each gene may present various disease-causing mutations leading to the same phenotype. Secondly, the genetic factor: different genes, when mutated, might induce the same pathological phenotype. Thirdly, the phenotypic factor: different mutations in the same gene can lead to different clinical pictures. Fourthly, the clinical factor: the same gene mutation can cause different signs and symptoms, even in members of the same family [40].

We analyzed the case of an undefined CORD affected family members showing a wide range of phenotypes. Such clinical heterogeneity could result from a combination of previously described factors. WES and WGS [41] permitted us to investigate the whole genome of the proband and find new potentially CORD-associated gene variants, which were then studied in the other family members. Such approach permitted to underline the multigenic nature of this group of retinal diseases, as well as the possible involvement of modifier genes into related phenotype heterogeneity.

The causative gene identified after all analyses resulted the *GUCY2D* (HGNC ID: 4689, OMIM: 600179) which encodes a retina-specific guanylate cyclase GC-E/RetGC1, which is highly expressed in the outer segment membranes of cones, and to a lesser extent in rods [42, 43]. GC-E is a key enzyme in the phototransduction cascade, catalyzing the production of cGMP (cyclic guanosine monophosphate) from GTP (guanosine triphosphate) in a calcium-sensitive manner [44, 45].

One variant found within *GUCY2D* gene, c.2513G>C, is already known to cause an autosomal dominant form of cone-rod dystrophy [46–48]. The combination of this variant with the c.3044-7G>T, also in *GUCY2D*, might enforce the effects of GUCY2D mutated form.

However, the presence of very heterogeneous phenotype exhibited by all affected family members made us assume that such complexity might be determined not only by the combination of previous cited variants, but also by mutated modifiers genes. Thus, by applying previously described filtering criteria and combining data mining with a deep eye-related pathway analysis, nine variants, carried by six genes (*PAX2*, *RXRG*, *CACNG8*, *CCDC175*, *PDE4DIP*, and *LTF*), emerged as potentially associated to the different phenotypes.

Our exploratory working hypothesis, explained below, starts from the primary phototransduction impairment determined by mutated GUCY2D, and bases its novelty on the recent discovery regarding photosensitive retinal ganglion cells (ipRGCs), and their capacity of expressing melanopsin intrinsically [49]. These cells represent the third class of retinal photoreceptors whose axon collaterals constitute a centrifugal pathway to upstream dopaminergic amacrine cells (DACs) forwarding melanopsin-based signals from the innermost retina to the outer retina [50]. Melanopsin-based membrane depolarization could trigger glutamate release locally onto DACs, through activation of AMPA glutamate receptors, depolarizing DACs and increasing action potential firing frequency, triggering dopamine release [50]. Increased dopamine release mediated by ipRGC activity (which integrates light-adapted cone and melanopsin signals) can also act through D4 dopamine receptors expressed on cones. Cones are known to affect cGMP metabolism, gene expression, and rod-cone coupling, regulating cone photoreceptor adaptation. In addition, the loss of M1 subtype of ipRGCs attenuates light adaptation, as evidenced by an impaired electroretinogram b-wave from cones [50].

This scenario highlights the central role of cones, whose impairment is primary at disease onset in all affected family members, as evidenced by clinical exams and by altered color perception. Such cone compromised activity, originated from GUCY2Ddefects, might be differentially worsened by altered retrograde signaling from the inner retinal cells due to variants carried by candidate modifier genes.

The driver modifier activity might belong to c.*6819A>T carried by *CACNG8*, which encodes the γ-8 member of transmembrane AMPA receptor-associated regulatory proteins (TARPs). TARPs control synaptic strength both by targeting AMPARs to synapses through an intracellular PDZ-binding motif and by regulating their gating through an extracellular domain [51]. Although the extracellular domain of γ-8 can determine slower AMPAR decay kinetics, it is constrained by an inhibitory influence of AMPAR channel gating exerted by the intracellular domains of γ-8 [52].As previously reported, the frequency of *CACNG8* c.*6819A>Tis extremely low in the Messina population. This variant is candidate to decrease the expression of TARP γ-8 physiologically, interacting with DACs and RGCs AMPARs and reducing both retrograde and anterograde signal transmissions. The arrest of the anterograde pathway may send a stop signal to RGCs, impairing optic nerve activity, (evidenced in II1 and II4) by altered evoked potentials. Interruption of retrograde signaling may involve cones, which consequently may die, due to inability to perform neuronal transmission function.

Additionally, the presence of the *RXRG* splicing variant c.875+6A>G, not frequent in the Messina population, may also alter the phenotypic proportion of L and S cones and their differentiation from birth, leading to confused color perception. Such hypothesis is supported by the well-established role of RXRG in cone differentiation, mediated by 9-cis retinoic acid [53, 54].

An altered expression of *PAX2* c.-375C>A could also decrease cone cell genesis and correct glial phenotype manifestation. That is a consequence of impairing astrocytes and other neural supporting cells, especially in RPE, preventing it from ensuring a trophic and protective role towards the underlying photoreceptor layer of the retina [55–58].

This scenario could promote cone and rod death, due to the lack of fundamental anti-inflammatory activity of lactotransferrin [59, 60], which failed because of the presence of *LTF* c.85G>A and c.140A>G and, probably, *CCDC175* c.-79C>G and c.76 C>T.

Explaining the role of CCDC175, however, remains the most challenge aspect to be faced. Little is known about *CCDC175* (coiled-coil domain containing 175, 14q23.1), especially in homo sapiens. The only available data come from the STRING database and highlight an interaction between the *Mus musculus* ccdc175 protein and IL23R (Interleukin 23 Receptor), which is associated with two eye-relevant and rare diseases, Behçet’s Disease and Vogt-Koyanagi-Harada Syndrome [61–63]. We conducted a whole transcriptome analysis on human RPE cells which showed a decreased expression of *CCDC175*, after an oxidant chemical compound treatment. Therefore, we can say that expression of *CCDC175* occurs in the human retina, as well as other considered genes, established by the same RNA–Seq experiment (submitted data, partially shown in S3 Table).

**Fig 9:**
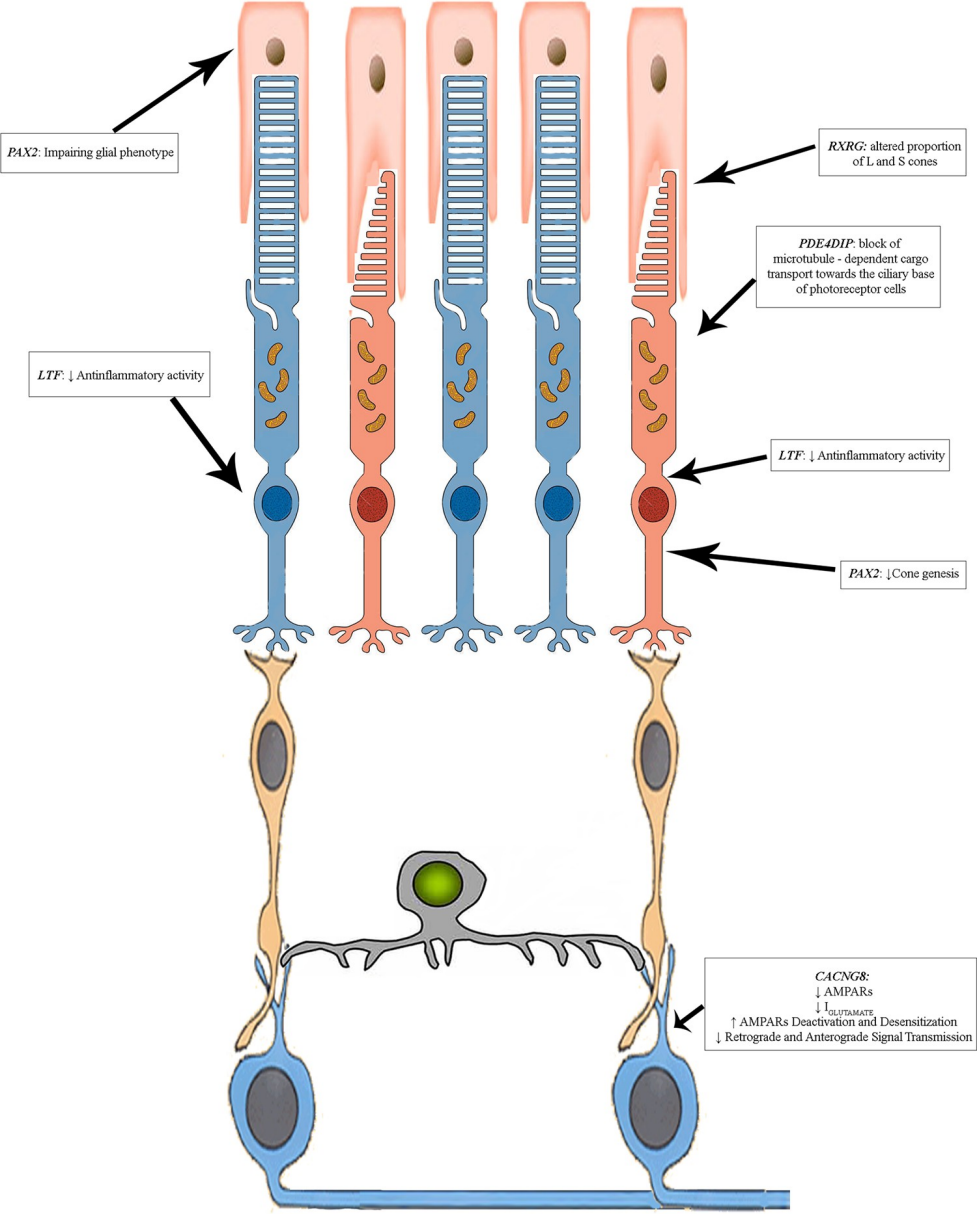
Proposed regulatory pattern related to CORD pathogenic mechanisms. Our hypothesis is based on primary cone compromised activity, originated from GUCY2D defects, might worsened by altered retrograde signaling from the inner retinal cells due to variants carried by candidate modifier genes. CACNG8 c.*6819A>T could decrease the expression of TARP γ-8 physiologically interacting with DACs and RGCs AMPARs, reducing the retrograde and anterograde signal transmission determining, respectively, an impairment of optic nerve activity and co death. Additionally, the presence of the RXRG c.875+6A>G and PAX2 c.-375C>A may have already compromised cones and their differentiation, probably involving astrocytes and other neural supporting cells, especially in RPE. This scenario could also promote rods death, due to the lack of the fundamental anti-inflammatory activity of lactotransferrin (failed because of LTF c.85G>A and c.140A>G and, probably, of CCDC175 c.-79C>G and c.76 C>T), and due to the reduced expression of PDE4DIP (caused by the two 3’ UTR variants c.*39G>A and c.*105 G>A), which could prevent the proper transport of phototransduction proteins between the inner and the outer photoreceptor segments. More details in the text.

Finally, photoreceptor death may be due to reduced expression of *PDE4DIP* variants, the 3’ UTR variants c.*39G>A and c.*105 G>A. These variants might determine the block of microtubule polymerization, preventing the proper transport of phototransduction proteins between the inner and the outer photoreceptor segments, mediated by USH transport network [64–68]. The hypothetical compromised functional scenario is represented in Fig 9. This pathophysiological model appears consistent with bioinformatic and literature data and is currently being experimentally tested.

## Conclusions

We identified a complex set of candidate gene-variants closely associated with a form of CORD mainly determined by the consequences of mutated *GUCY2D*. These candidate genes encode several fundamental proteins involved in retina and eye physiology, and might act as modifier genes determining the heterogeneity of exhibited phenotypes.

Our discovery could permit to progress towards a better classification of all CORD forms and to shed light on the mechanisms of the disease group.

However, our pilot exploratory study presented several limitations, such as the restricted number of examined patients and the absence of functional essays needed to confirm the obtained results about genotypic and phenotypic association. Furthermore, the additive model evaluated is not warranted, and most interesting data came from WES data only. Additionally, even if important symptomatology differences are certain for affected members, others might be reduced during the disease course. Thus, next step will foresee experimental validation (e. g. protein–protein interaction assay by NanoBiT^®^ and electrophysiological functional assays) of analyzed data to give our findings more strength, as well as the involvement of a wider cohort of patients with genotype—phenotype correlation similar to our examined family.

In this way, our results could provide additional prognostic clues for genetic counseling and a better molecular characterization of patients, allowing them to be included in future clinical trials based on gene therapy.

## Supporting information

S1 Fig Autofluorescence, optical coherence tomography (OCT) and electroretinogram (ERG) of patient II1. Oval hypo-AF foveal lesions were evident in both eyes (a, e), as well as a transverse loss of the junction between the inner and outer segment of the photoreceptors in the foveal region (b, f). Both scotopic (c, g) and photopic (d, h) ERGs resulted almost extinct. Fundus photo of left eye is shown upside down.
(PDF)

S2 Fig Fundus, optical coherence tomography (OCT) and electroretinogram (ERG) of patient II2. Fundus examination highlighted slightly pale optic discs with almost normal retinal vessels, pigment accumulation and moderate bone-like spicules in peripheral areas (a, e). OCT scans revealed a compromission of the junction between the inner and outer segment of the photoreceptors in the foveal region (b, f). Scotopic (c, g) and photopic (d, h) ERGs resulted both extinct. Fundus photo of left eye is shown upside down.
(PDF)

S3 Fig Autofluorescence, optical coherence tomography (OCT) and electroretinogram (ERG) of patient II4. Serious AF foveal lesions were evident in both eyes (a, e), as well as a transverse loss of the junction between the inner and outer segment of the photoreceptors in the foveal region (b, f). Both scotopic (c, g) and photopic (d, h) ERGs resulted almost extinct. Fundus photo of left eye is shown upside down.
(PDF)

S4 Fig Cytoscape preliminary pathway analysis. The figure shows the Cytoscape preliminary pathway analysis, supported by GeneMANIA plug-in, with nodes and edges reflecting genetic (a) and physical (b) relationships between query genes. *CACNG8* appears as the most genetic perturbing gene, while *RXRG* and *PDE4DIP* emerge as the most physical interacting genes.
(PDF)

S5 Fig CluePedia enrichment analysis of 10 best candidate markers. Based on publicly available data from STRING and mIRANDA, 93100 terms were connected by 247 edges, related to enrichment categories: activation (green), binding (blue), catalysis (deep purple), expression (yellow), inhibition (red), ptmod (light purple), reaction (black). Only relevant enrichment (*p* < 0.05) in the identified protein interactome is shown.
(PDF)

S6 Fig CluePedia “Cerebral layout”. Most of the proteins encoded by the 6 candidate genes are located in intracellular compartments with only one (LTF) extracellular and one (CACNG8) located in the plasma membrane.
(PDF)

S7 Fig CluePedia mIRANDA network enrichment. mIRANDA database showed that 2–9 miRNAs could regulate the expression of each analyzed gene.
(PDF)

S8 Fig Genotype of variants. For each subject, genotypes of all analyzed variants are shown on a row of three heatmaps that are built according to an additive (a), dominant (b), or recessive (c) model, respectively. Each column thus represents the genotype of a given variant. In the additive model (a), yellow pixels correspond to wildtype, red ones to heterozygous mutation, and green pixels show mutations in homozygosis. In the dominant (b) and recessive models (c), red pixels highlight variants which were not considered as being mutated, whereas blue ones mean that, under the model considered, mutation occurred.
(PDF)

S9 Fig Genotype—phenotype correlations. a) Genotypes distribution of each subject was compared with those of all the other subjects. Such analysis resulted in a score ranging between 0 (blue) and 1 (yellow); the higher this value, the more similar genotypes distribution between two given subjects. b) By applying a cutoff to such maps at a given percentage, only pixels corresponding to subject pairs whose genotype distributions were more similar persist; here a representative similarity cutoff of 70% was chosen. c) The same correspondence could be obtained by considering symptom patterns. After applying a strong cutoff (80% in this case), subjects showing a close correspondence in symptoms distribution are highlighted (d).
(PDF)

S10 Fig Frequency distribution of candidate variants through the population of Messina. As evidenced by bar plots (a-i), at least two variants, *CACNG8* rs66507429:A>T (a) and *CCDC175*c.76 C>T (e), show a very low frequency distribution in healthy population, suggesting a possible association with pathology.
(PDF)

S1 Table ASSP output table for *GUCY2D* c.3044-7G>T. Tables show the results from ASSP prediction analyses for both wild-type and c.3044-7G>T carrying *GUCY2D*.
(XLSX)

S2 Table ClueGO preliminary pathway analysis involving the 6 candidate genes. The Cytoscape ClueGO plug-in permitted us to cluster selected candidate genes into common pathways, and detailed results are shown in this table. GO-ID: ID associated to Gene Ontology. GO-Term: specific GO annotation. Ontology Source: databases from which was derived the annotation. Term p-value: statistical significance of predicted annotation. Term p-value Corrected with Bonferroni step down: statistical correction applied to previous statistical association analysis. Group p-value: statistical significance of predicted clustered annotations. Group p-value Corrected with Bonferroni step down: statistical correction applied to previous statistical clustering analysis. GO-Levels: Due to the complex structure of GO tree (directed acyclic graph), the GO terms were placed in several levels. In case of using sources without hierarchical structure (KEGG, REACTOME, WikiPathways), the level it is assigned as -1. GO-Groups: the group or the groups that include the term. % Associated Genes: percentage of the genes from the uploaded cluster that were associated with the term, compared with all the genes associated with the term. Nr. Genes: number of the genes from the uploaded cluster that were associated with the term. Gene Symbols: symbols of genes clustered in the same group.
(XLSX)

S3 Table RNA–Seq experiment on RPE cells established *PDE4DIP*, *PAX2*, *LTF*, *RXRG*, *CACNG8* and *CCDC175* expression in the retina. A whole transcriptome analysis realized on RPE cells, treated with oxidant compounds versus untreated, (full data not shown) highlighted the expression changes of *PDE4DIP*, *PAX2*, *LTF*, *RXRG*, *CACNG8* and *CCDC175* genes in human retina. Fold–change 1h: expression difference between treated and untreated samples after 1h. Fold–change 2h: expression difference between treated and untreated samples after 2h. Fold–change 4h: expression difference between treated and untreated samples after 4h. Fold–change 1h: expression difference between treated and untreated samples after 6h. GO biological process, GO cellular component and GO molecular function: annotations of selected gene by Gene Ontology main categories. Database object name: extended name of analyzed gene.
(XLSX)

## References

[1] Gill JS, Georgiou M, Kalitzeos A, Moore AT, Michaelides M. Progressive cone and cone-rod dystrophies: clinical features, molecular genetics and prospects for therapy. The British journal of ophthalmology. 2019. Epub 20190124. doi: 10.1136/bjophthalmol-2018-313278 ; PubMed Central PMCID: PMC6709772. 30679166 PMC6709772

[2] Liew G, Michaelides M, Bunce C. A comparison of the causes of blindness certifications in England and Wales in working age adults (16–64 years), 1999–2000 with 2009–2010. BMJ Open. 2014;4(2):e004015. Epub 20140212. doi: 10.1136/bmjopen-2013-004015 ; PubMed Central PMCID: PMC3927710. 24525390 PMC3927710

[3] Tsang SH, Sharma T. Progressive Cone Dystrophy and Cone-Rod Dystrophy (XL, AD, and AR). Adv Exp Med Biol. 2018;1085:53–60. doi: 10.1007/978-3-319-95046-4_12 . 30578485

[4] Bolz HJ. [Genetic diagnostics of retinal dystrophies: Breakthrough with new methods of DNA sequencing]. Ophthalmologe. 2018;115(12):1028–34. doi: 10.1007/s00347-018-0762-5 . 30054723

[5] Ke R, Mignardi M, Hauling T, Nilsson M. Fourth Generation of Next-Generation Sequencing Technologies: Promise and Consequences. Hum Mutat. 2016;37(12):1363–7. doi: 10.1002/humu.23051 ; PubMed Central PMCID: PMC5111608. 27406789 PMC5111608

[6] Perez-Carro R, Corton M, Sanchez-Navarro I, Zurita O, Sanchez-Bolivar N, Sanchez-Alcudia R, et al. Panel-based NGS Reveals Novel Pathogenic Mutations in Autosomal Recessive Retinitis Pigmentosa. Sci Rep. 2016;6:19531. doi: 10.1038/srep19531 ; PubMed Central PMCID: PMC4726392. 26806561 PMC4726392

[7] CLC Genomics Workbench 20.0 2019 [cited 2020 April 2020]. Available from: https://digitalinsights.qiagen.com.

[8] Wang K, Li M, Hakonarson H. ANNOVAR: functional annotation of genetic variants from high-throughput sequencing data. Nucleic Acids Res. 2010;38(16):e164. Epub 20100703. doi: 10.1093/nar/gkq603 ; PubMed Central PMCID: PMC2938201. 20601685 PMC2938201

[9] Stenson PD, Ball EV, Mort M, Phillips AD, Shiel JA, Thomas NS, et al. Human Gene Mutation Database (HGMD): 2003 update. Hum Mutat. 2003;21(6):577–81. Epub 2003/05/20. doi: 10.1002/humu.10212 . 12754702

[10] Koressaar T, Lepamets M, Kaplinski L, Raime K, Andreson R, Remm M. Primer3_masker: integrating masking of template sequence with primer design software. Bioinformatics. 2018;34(11):1937–8. Epub 2018/01/24. doi: 10.1093/bioinformatics/bty036 . 29360956

[11] Shannon P, Markiel A, Ozier O, Baliga NS, Wang JT, Ramage D, et al. Cytoscape: a software environment for integrated models of biomolecular interaction networks. Genome Res. 2003;13(11):2498–504. doi: 10.1101/gr.1239303 ; PubMed Central PMCID: PMC403769. 14597658 PMC403769

[12] Warde-Farley D, Donaldson SL, Comes O, Zuberi K, Badrawi R, Chao P, et al. The GeneMANIA prediction server: biological network integration for gene prioritization and predicting gene function. Nucleic Acids Res. 2010;38(Web Server issue):W214-20. doi: 10.1093/nar/gkq537 ; PubMed Central PMCID: PMC2896186. 20576703 PMC2896186

[13] Bindea G, Galon J, Mlecnik B. CluePedia Cytoscape plugin: pathway insights using integrated experimental and in silico data. Bioinformatics. 2013;29(5):661–3. doi: 10.1093/bioinformatics/btt019 ; PubMed Central PMCID: PMC3582273. 23325622 PMC3582273

[14] Szklarczyk D, Franceschini A, Wyder S, Forslund K, Heller D, Huerta-Cepas J, et al. STRING v10: protein-protein interaction networks, integrated over the tree of life. Nucleic Acids Res. 2015;43(Database issue):D447–52. doi: 10.1093/nar/gku1003 ; PubMed Central PMCID: PMC4383874. 25352553 PMC4383874

[15] Orchard S, Ammari M, Aranda B, Breuza L, Briganti L, Broackes-Carter F, et al. The MIntAct project—IntAct as a common curation platform for 11 molecular interaction databases. Nucleic Acids Res. 2014;42(Database issue):D358–63. doi: 10.1093/nar/gkt1115 ; PubMed Central PMCID: PMC3965093. 24234451 PMC3965093

[16] Tarcea VG, Weymouth T, Ade A, Bookvich A, Gao J, Mahavisno V, et al. Michigan molecular interactions r2: from interacting proteins to pathways. Nucleic Acids Res. 2009;37(Database issue):D642–6. doi: 10.1093/nar/gkn722 ; PubMed Central PMCID: PMC2686565. 18978014 PMC2686565

[17] Kozomara A, Griffiths-Jones S. miRBase: annotating high confidence microRNAs using deep sequencing data. Nucleic Acids Res. 2014;42(Database issue):D68–73. doi: 10.1093/nar/gkt1181 ; PubMed Central PMCID: PMC3965103. 24275495 PMC3965103

[18] Xiao F, Zuo Z, Cai G, Kang S, Gao X, Li T. miRecords: an integrated resource for microRNA-target interactions. Nucleic Acids Res. 2009;37(Database issue):D105–10. doi: 10.1093/nar/gkn851 ; PubMed Central PMCID: PMC2686554. 18996891 PMC2686554

[19] Bhattacharya A, Ziebarth JD, Cui Y. PolymiRTS Database 3.0: linking polymorphisms in microRNAs and their target sites with human diseases and biological pathways. Nucleic Acids Res. 2014;42(Database issue):D86–91. doi: 10.1093/nar/gkt1028 ; PubMed Central PMCID: PMC3965097. 24163105 PMC3965097

[20] Birtel J, Eisenberger T, Gliem M, Muller PL, Herrmann P, Betz C, et al. Clinical and genetic characteristics of 251 consecutive patients with macular and cone/cone-rod dystrophy. Sci Rep. 2018;8(1):4824. Epub 20180319. doi: 10.1038/s41598-018-22096-0 ; PubMed Central PMCID: PMC5859282. 29555955 PMC5859282

[21] Duncker T, Tsang SH, Lee W, Zernant J, Allikmets R, Delori FC, et al. Quantitative fundus autofluorescence distinguishes ABCA4-associated and non-ABCA4-associated bull’s-eye maculopathy. Ophthalmology. 2015;122(2):345–55. Epub 2014/10/07. doi: 10.1016/j.ophtha.2014.08.017 ; PubMed Central PMCID: PMC4306619. 25283059 PMC4306619

[22] Ito S, Nakamura M, Ohnishi Y, Miyake Y. Autosomal dominant cone-rod dystrophy with R838H and R838C mutations in the GUCY2D gene in Japanese patients. Jpn J Ophthalmol. 2004;48(3):228–35. Epub 2004/06/04. doi: 10.1007/s10384-003-0050-y . 15175914

[23] Jiang F, Xu K, Zhang X, Xie Y, Bai F, Li Y. GUCY2D mutations in a Chinese cohort with autosomal dominant cone or cone-rod dystrophies. Doc Ophthalmol. 2015;131(2):105–14. Epub 2015/08/25. doi: 10.1007/s10633-015-9509-7 . 26298565

[24] Kohl S, Kitiratschky V, Papke M, Schaich S, Sauer A, Wissinger B. Genes and mutations in autosomal dominant cone and cone-rod dystrophy. Adv Exp Med Biol. 2012;723:337–43. Epub 2011/12/21. doi: 10.1007/978-1-4614-0631-0_44 . 22183351

[25] Liu X, Fujinami K, Kuniyoshi K, Kondo M, Ueno S, Hayashi T, et al. Clinical and Genetic Characteristics of 15 Affected Patients From 12 Japanese Families with GUCY2D-Associated Retinal Disorder. Transl Vis Sci Technol. 2020;9(6):2. Epub 2020/08/22. doi: 10.1167/tvst.9.6.2 ; PubMed Central PMCID: PMC7408927. 32821499 PMC7408927

[26] Mukherjee R, Robson AG, Holder GE, Stockman A, Egan CA, Moore AT, et al. A detailed phenotypic description of autosomal dominant cone dystrophy due to a de novo mutation in the GUCY2D gene. Eye (Lond). 2014;28(4):481–7. Epub 2014/02/01. doi: 10.1038/eye.2014.7 ; PubMed Central PMCID: PMC3983649. 24480840 PMC3983649

[27] Payne AM, Morris AG, Downes SM, Johnson S, Bird AC, Moore AT, et al. Clustering and frequency of mutations in the retinal guanylate cyclase (GUCY2D) gene in patients with dominant cone-rod dystrophies. J Med Genet. 2001;38(9):611–4. Epub 2001/09/22. doi: 10.1136/jmg.38.9.611 ; PubMed Central PMCID: PMC1734946. 11565546 PMC1734946

[28] Riera M, Navarro R, Ruiz-Nogales S, Mendez P, Bures-Jelstrup A, Corcostegui B, et al. Whole exome sequencing using Ion Proton system enables reliable genetic diagnosis of inherited retinal dystrophies. Sci Rep. 2017;7:42078. Epub 2017/02/10. doi: 10.1038/srep42078 ; PubMed Central PMCID: PMC5299602. 28181551 PMC5299602

[29] Rodriguez-Munoz A, Aller E, Jaijo T, Gonzalez-Garcia E, Cabrera-Peset A, Gallego-Pinazo R, et al. Expanding the Clinical and Molecular Heterogeneity of Nonsyndromic Inherited Retinal Dystrophies. J Mol Diagn. 2020;22(4):532–43. Epub 2020/02/10. doi: 10.1016/j.jmoldx.2020.01.003 . 32036094

[30] Shanks ME, Downes SM, Copley RR, Lise S, Broxholme J, Hudspith KA, et al. Next-generation sequencing (NGS) as a diagnostic tool for retinal degeneration reveals a much higher detection rate in early-onset disease. Eur J Hum Genet. 2013;21(3):274–80. Epub 2012/09/13. doi: 10.1038/ejhg.2012.172 ; PubMed Central PMCID: PMC3573204. 22968130 PMC3573204

[31] Sharon D, Ben-Yosef T, Goldenberg-Cohen N, Pras E, Gradstein L, Soudry S, et al. A nationwide genetic analysis of inherited retinal diseases in Israel as assessed by the Israeli inherited retinal disease consortium (IIRDC). Hum Mutat. 2020;41(1):140–9. Epub 2019/08/29. doi: 10.1002/humu.23903 . 31456290

[32] Turro E, Astle WJ, Megy K, Graf S, Greene D, Shamardina O, et al. Whole-genome sequencing of patients with rare diseases in a national health system. Nature. 2020;583(7814):96–102. Epub 2020/06/26. doi: 10.1038/s41586-020-2434-2 ; PubMed Central PMCID: PMC7610553. 32581362 PMC7610553

[33] Udar N, Yelchits S, Chalukya M, Yellore V, Nusinowitz S, Silva-Garcia R, et al. Identification of GUCY2D gene mutations in CORD5 families and evidence of incomplete penetrance. Hum Mutat. 2003;21(2):170–1. Epub 2003/01/29. doi: 10.1002/humu.9109 . 12552567

[34] Wilkie SE, Newbold RJ, Deery E, Walker CE, Stinton I, Ramamurthy V, et al. Functional characterization of missense mutations at codon 838 in retinal guanylate cyclase correlates with disease severity in patients with autosomal dominant cone-rod dystrophy. Human molecular genetics. 2000;9(20):3065–73. Epub 2000/12/15. doi: 10.1093/hmg/9.20.3065 . 11115851

[35] Xiao X, Guo X, Jia X, Li S, Wang P, Zhang Q. A recurrent mutation in GUCY2D associated with autosomal dominant cone dystrophy in a Chinese family. Mol Vis. 2011;17:3271–8. Epub 2011/12/24. ; PubMed Central PMCID: PMC3244478. 22194653 PMC3244478

[36] Zobor D, Zrenner E, Wissinger B, Kohl S, Jagle H. GUCY2D- or GUCA1A-related autosomal dominant cone-rod dystrophy: is there a phenotypic difference? Retina. 2014;34(8):1576–87. Epub 2014/05/31. doi: 10.1097/IAE.0000000000000129 . 24875811

[37] Barsky A, Gardy JL, Hancock RE, Munzner T. Cerebral: a Cytoscape plugin for layout of and interaction with biological networks using subcellular localization annotation. Bioinformatics. 2007;23(8):1040–2. doi: 10.1093/bioinformatics/btm057 . 17309895

[38] Donato L, Bramanti P, Scimone C, Rinaldi C, D’Angelo R, Sidoti A. miRNAexpression profile of retinal pigment epithelial cells under oxidative stress conditions. FEBS Open Bio. 2018;8(2):219–33. Epub 2018/02/13. doi: 10.1002/2211-5463.12360 ; PubMed Central PMCID: PMC5794457. 29435412 PMC5794457

[39] Donato L, Scimone C, Alibrandi S, Rinaldi C, Sidoti A, D’Angelo R. Transcriptome Analyses of lncRNAs in A2E-Stressed Retinal Epithelial Cells Unveil Advanced Links between Metabolic Impairments Related to Oxidative Stress and Retinitis Pigmentosa. Antioxidants (Basel). 2020;9(4). Epub 2020/04/25. doi: 10.3390/antiox9040318 . 32326576 PMC7222347

[40] Reiff C, Owczarek-Lipska M, Spital G, Roger C, Hinz H, Juschke C, et al. The mutation p.E113K in the Schiff base counterion of rhodopsin is associated with two distinct retinal phenotypes within the same family. Sci Rep. 2016;6:36208. doi: 10.1038/srep36208 ; PubMed Central PMCID: PMC5095885. 27812022 PMC5095885

[41] Park ST, Kim J. Trends in Next-Generation Sequencing and a New Era for Whole Genome Sequencing. Int Neurourol J. 2016;20(Suppl 2):S76–83. doi: 10.5213/inj.1632742.371 ; PubMed Central PMCID: PMC5169091. 27915479 PMC5169091

[42] Dizhoor AM, Lowe DG, Olshevskaya EV, Laura RP, Hurley JB. The human photoreceptor membrane guanylyl cyclase, RetGC, is present in outer segments and is regulated by calcium and a soluble activator. Neuron. 1994;12(6):1345–52. doi: 10.1016/0896-6273(94)90449-9 . 7912093

[43] Liu X, Seno K, Nishizawa Y, Hayashi F, Yamazaki A, Matsumoto H, et al. Ultrastructural localization of retinal guanylate cyclase in human and monkey retinas. Exp Eye Res. 1994;59(6):761–8. doi: 10.1006/exer.1994.1162 . 7698269

[44] Peshenko IV, Moiseyev GP, Olshevskaya EV, Dizhoor AM. Factors that determine Ca2+ sensitivity of photoreceptor guanylyl cyclase. Kinetic analysis of the interaction between the Ca2+-bound and the Ca2+-free guanylyl cyclase activating proteins (GCAPs) and recombinant photoreceptor guanylyl cyclase 1 (RetGC-1). Biochemistry. 2004;43(43):13796–804. doi: 10.1021/bi048943m . 15504042

[45] Shyjan AW, de Sauvage FJ, Gillett NA, Goeddel DV, Lowe DG. Molecular cloning of a retina-specific membrane guanylyl cyclase. Neuron. 1992;9(4):727–37. doi: 10.1016/0896-6273(92)90035-c . 1356371

[46] Auz-Alexandre CL, Vallespin E, Aguirre-Lamban J, Cantalapiedra D, Avila-Fernandez A, Villaverde-Montero C, et al. Novel human pathological mutations. Gene symbol: GUCY2D. Disease: Leber congenital amaurosis. Human genetics. 2009;125(3):349. . 19320033

[47] Garcia-Hoyos M, Auz-Alexandre CL, Almoguera B, Cantalapiedra D, Riveiro-Alvarez R, Lopez-Martinez MA, et al. Mutation analysis at codon 838 of the Guanylate Cyclase 2D gene in Spanish families with autosomal dominant cone, cone-rod, and macular dystrophies. Mol Vis. 2011;17:1103–9. Epub 20110429. ; PubMed Central PMCID: PMC3087450. 21552474 PMC3087450

[48] Xu F, Dong F, Li H, Li X, Jiang R, Sui R. Phenotypic characterization of a Chinese family with autosomal dominant cone-rod dystrophy related to GUCY2D. Doc Ophthalmol. 2013;126(3):233–40. Epub 20130521. doi: 10.1007/s10633-013-9383-0 . 23686677

[49] Zhang J, Wang H, Wu S, Liu Q, Wang N. Regulation of Reentrainment Function Is Dependent on a Certain Minimal Number of Intact Functional ipRGCs in rd Mice. J Ophthalmol. 2017;2017:6804853. Epub 2018/01/24. doi: 10.1155/2017/6804853 ; PubMed Central PMCID: PMC5735630. 29359039 PMC5735630

[50] Prigge CL, Yeh PT, Liou NF, Lee CC, You SF, Liu LL, et al. M1 ipRGCs Influence Visual Function through Retrograde Signaling in the Retina. J Neurosci. 2016;36(27):7184–97. doi: 10.1523/JNEUROSCI.3500-15.2016 ; PubMed Central PMCID: PMC4938862. 27383593 PMC4938862

[51] Sumioka A, Brown TE, Kato AS, Bredt DS, Kauer JA, Tomita S. PDZ binding of TARPgamma-8 controls synaptic transmission but not synaptic plasticity. Nat Neurosci. 2011;14(11):1410–2. doi: 10.1038/nn.2952 ; PubMed Central PMCID: PMC3206644. 22002768 PMC3206644

[52] Milstein AD, Nicoll RA. TARP modulation of synaptic AMPA receptor trafficking and gating depends on multiple intracellular domains. Proc Natl Acad Sci U S A. 2009;106(27):11348–51. doi: 10.1073/pnas.0905570106 ; PubMed Central PMCID: PMC2708767. 19549880 PMC2708767

[53] Onishi A, Peng GH, Chen S, Blackshaw S. Pias3-dependent SUMOylation controls mammalian cone photoreceptor differentiation. Nat Neurosci. 2010;13(9):1059–65. doi: 10.1038/nn.2618 ; PubMed Central PMCID: PMC2932661. 20729845 PMC2932661

[54] Swaroop A, Kim D, Forrest D. Transcriptional regulation of photoreceptor development and homeostasis in the mammalian retina. Nat Rev Neurosci. 2010;11(8):563–76. doi: 10.1038/nrn2880 . 20648062 PMC11346175

[55] Torres M, Gomez-Pardo E, Gruss P. Pax2 contributes to inner ear patterning and optic nerve trajectory. Development. 1996;122(11):3381–91. doi: 10.1242/dev.122.11.3381 . 8951055

[56] Fujimura N, Klimova L, Antosova B, Smolikova J, Machon O, Kozmik Z. Genetic interaction between Pax6 and beta-catenin in the developing retinal pigment epithelium. Dev Genes Evol. 2015;225(2):121–8. doi: 10.1007/s00427-015-0493-4 . 25689933

[57] Scimone C, Donato L, Alibrandi S, Vadala M, Giglia G, Sidoti A, et al. N-retinylidene-N-retinylethanolamine adduct induces expression of chronic inflammation cytokines in retinal pigment epithelium cells. Exp Eye Res. 2021;209:108641. Epub 20210529. doi: 10.1016/j.exer.2021.108641 . 34058230

[58] Rinaldi C, Donato L, Alibrandi S, Scimone C, D’Angelo R, Sidoti A. Oxidative Stress and the Neurovascular Unit. Life (Basel). 2021;11(8). Epub 20210729. doi: 10.3390/life11080767 ; PubMed Central PMCID: PMC8398978. 34440511 PMC8398978

[59] Moreno-Navarrete JM, Ortega F, Moreno M, Serrano M, Ricart W, Fernandez-Real JM. Lactoferrin gene knockdown leads to similar effects to iron chelation in human adipocytes. J Cell Mol Med. 2014;18(3):391–5. doi: 10.1111/jcmm.12234 ; PubMed Central PMCID: PMC3955146. 24571258 PMC3955146

[60] Araki-Sasaki K, Hirano K, Osakabe Y, Kuroda M, Kitagawa K, Mishima H, et al. Classification of secondary corneal amyloidosis and involvement of lactoferrin. Ophthalmology. 2013;120(6):1166–72. doi: 10.1016/j.ophtha.2012.11.047 . 23453509

[61] Cao S, Chee SP, Yu HG, Sukavatcharin S, Wu L, Kijlstra A, et al. Investigation of the association of Vogt-Koyanagi-Harada syndrome with IL23R-C1orf141 in Han Chinese Singaporean and ADO-ZNF365-EGR2 in Thai. The British journal of ophthalmology. 2016;100(3):436–42. doi: 10.1136/bjophthalmol-2015-307366 . 26628628

[62] Hou S, Liao D, Zhang J, Fang J, Chen L, Qi J, et al. Genetic variations of IL17F and IL23A show associations with Behcet’s disease and Vogt-Koyanagi-Harada syndrome. Ophthalmology. 2015;122(3):518–23. doi: 10.1016/j.ophtha.2014.09.025 . 25439430

[63] Yu H, Zheng M, Zhang L, Li H, Zhu Y, Cheng L, et al. Identification of susceptibility SNPs in IL10 and IL23R-IL12RB2 for Behcet’s disease in Han Chinese. J Allergy Clin Immunol. 2016. doi: 10.1016/j.jaci.2016.05.024 . 27464962

[64] Shapshak P. Molecule of the month, PDE4DIP. Bioinformation. 2012;8(16):740–1. doi: 10.6026/97320630008740 ; PubMed Central PMCID: PMC3449385. 23055623 PMC3449385

[65] Roubin R, Acquaviva C, Chevrier V, Sedjai F, Zyss D, Birnbaum D, et al. Myomegalin is necessary for the formation of centrosomal and Golgi-derived microtubules. Biol Open. 2013;2(2):238–50. doi: 10.1242/bio.20123392 ; PubMed Central PMCID: PMC3575658. 23430395 PMC3575658

[66] Di Gioia SA, Farinelli P, Letteboer SJ, Arsenijevic Y, Sharon D, Roepman R, et al. Interactome analysis reveals that FAM161A, deficient in recessive retinitis pigmentosa, is a component of the Golgi-centrosomal network. Human molecular genetics. 2015;24(12):3359–71. doi: 10.1093/hmg/ddv085 . 25749990

[67] Overlack N, Kilic D, Bauss K, Marker T, Kremer H, van Wijk E, et al. Direct interaction of the Usher syndrome 1G protein SANS and myomegalin in the retina. Biochimica et biophysica acta. 2011;1813(10):1883–92. doi: 10.1016/j.bbamcr.2011.05.015 . 21767579

[68] Donato L, Abdalla EM, Scimone C, Alibrandi S, Rinaldi C, Nabil KM, et al. Impairments of Photoreceptor Outer Segments Renewal and Phototransduction Due to a Peripherin Rare Haplotype Variant: Insights from Molecular Modeling. Int J Mol Sci. 2021;22(7). Epub 2021/04/04. doi: 10.3390/ijms22073484 . 33801777 PMC8036374

