# Supplementary figures and images for: The impact of modifier genes on cone-rod dystrophy heterogeneity: An explorative familial pilot study and a hypothesis on neurotransmission impairment
Source: PLoS One. 2022 Dec 9;17(12):e0278857. doi: 10.1371/journal.pone.0278857 (PMC9733859; doi:10.1371/journal.pone.0278857)

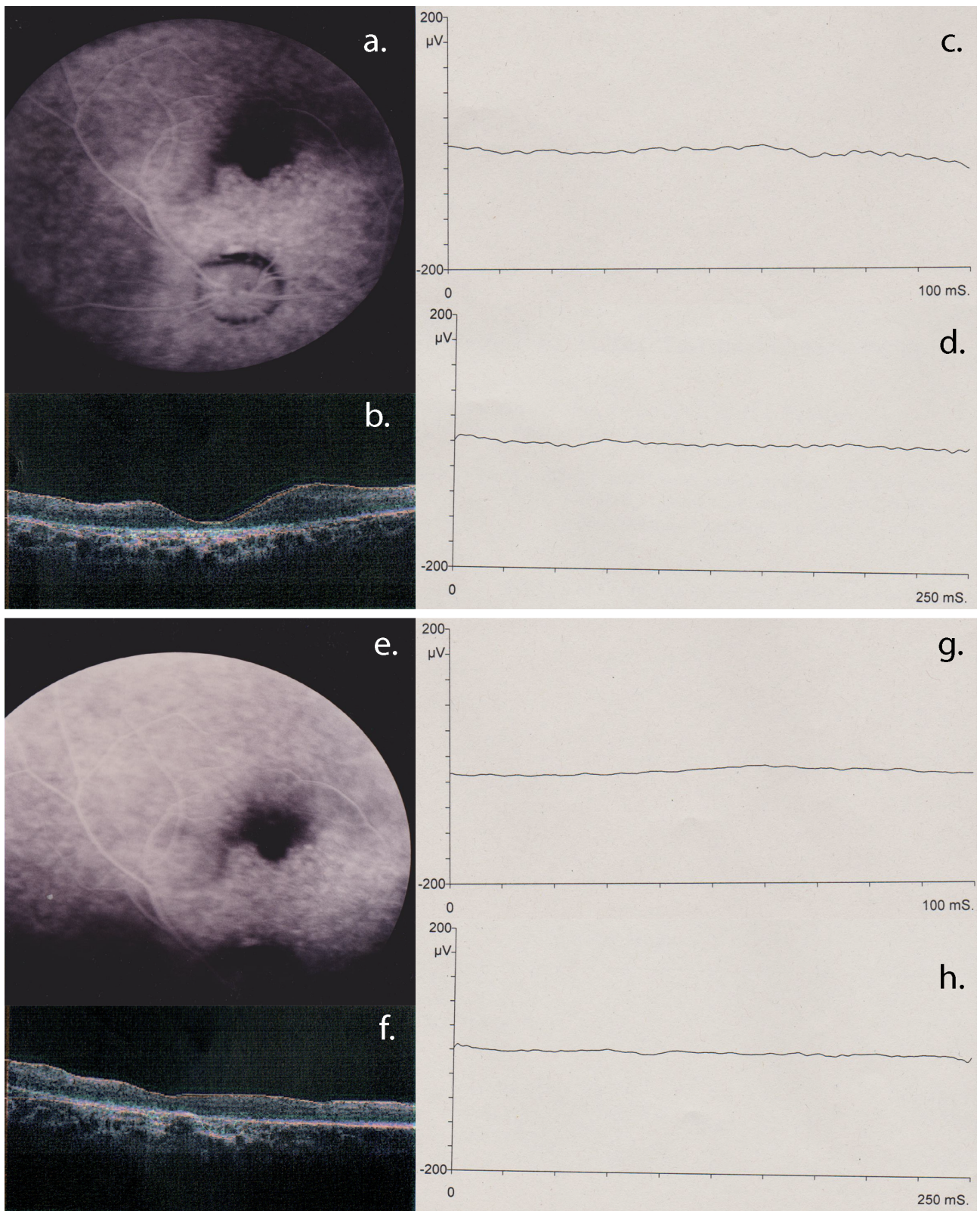

**S1 Fig**

Supplement: S1 Fig — Oval hypo-AF foveal lesions were evident in both eyes (a, e), as well as a transverse loss of the junction between the inner and outer segment of the photoreceptors in the foveal region (b, f). Both scotopic (c, g) and photopic (d, h) ERGs resulted almost extinct. Fundus photo of left eye is shown upside down. (PDF) [file pone.0278857.s001.pdf]

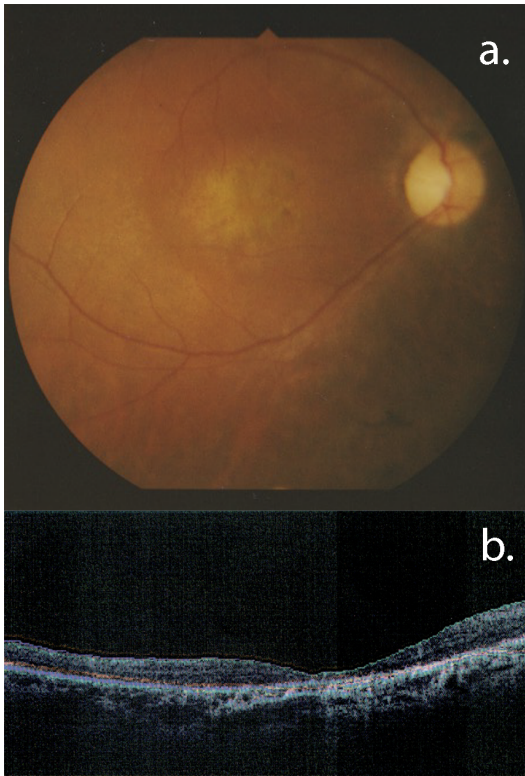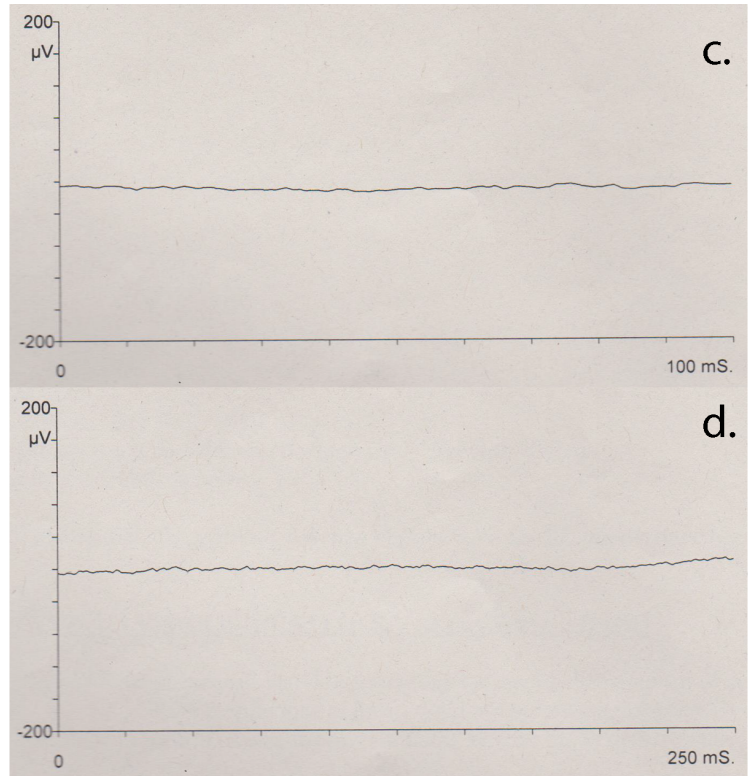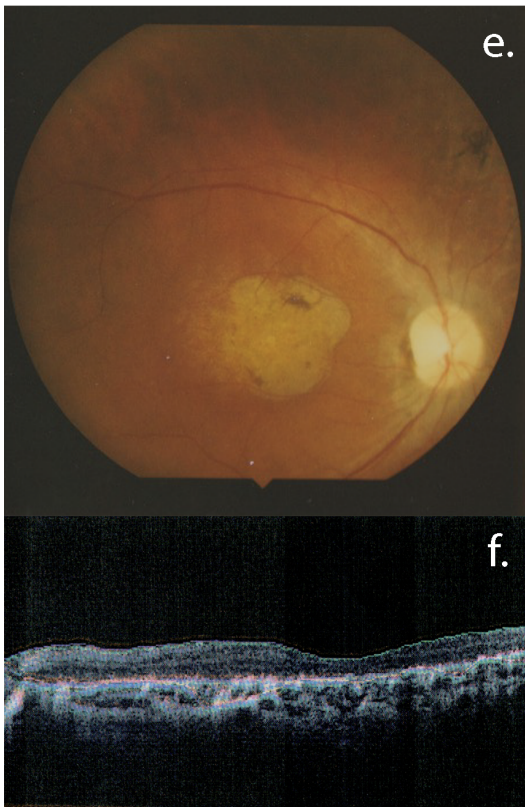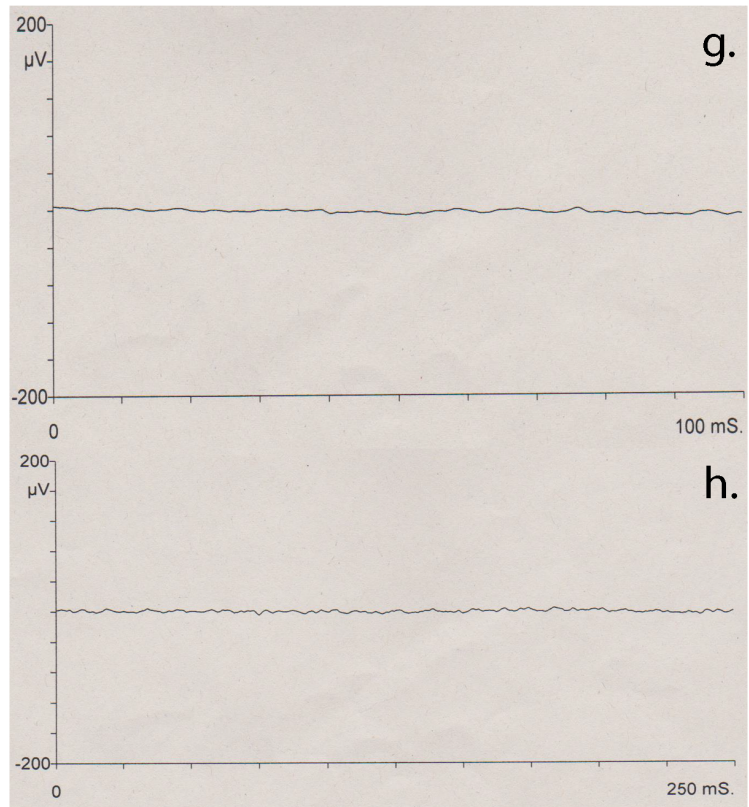

**S2 Fig**

Supplement: S2 Fig — Fundus examination highlighted slightly pale optic discs with almost normal retinal vessels, pigment accumulation and moderate bone-like spicules in peripheral areas (a, e). OCT scans revealed a compromission of the junction between the inner and outer segment of the photoreceptors in the foveal region (b, f). Scotopic (c, g) and photopic (d, h) ERGs resulted both extinct. Fundus photo of left eye is shown upside down. (PDF) [file pone.0278857.s002.pdf]

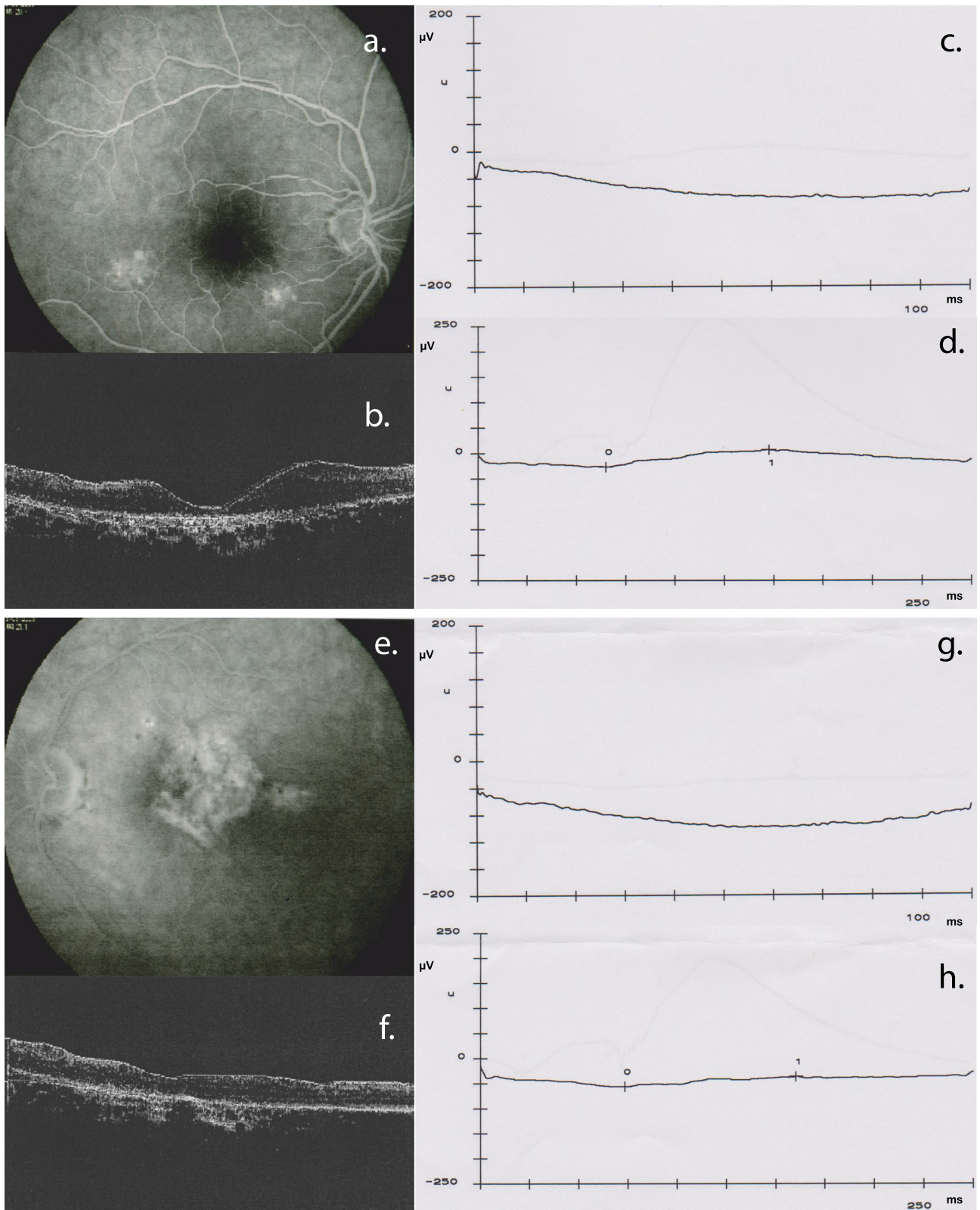

**S3 Fig**

Supplement: S3 Fig — Serious AF foveal lesions were evident in both eyes (a, e), as well as a transverse loss of the junction between the inner and outer segment of the photoreceptors in the foveal region (b, f). Both scotopic (c, g) and photopic (d, h) ERGs resulted almost extinct. Fundus photo of left eye is shown upside down. (PDF) [file pone.0278857.s003.pdf]

a.

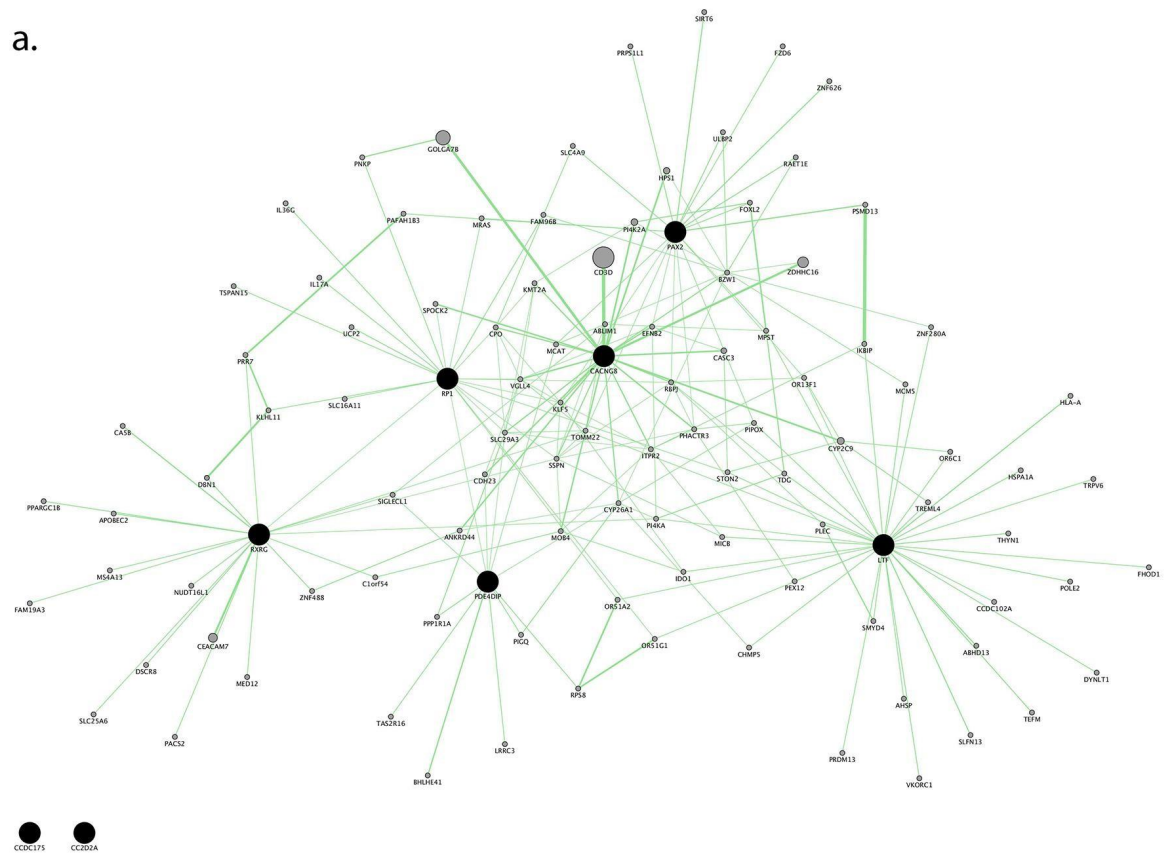

b.

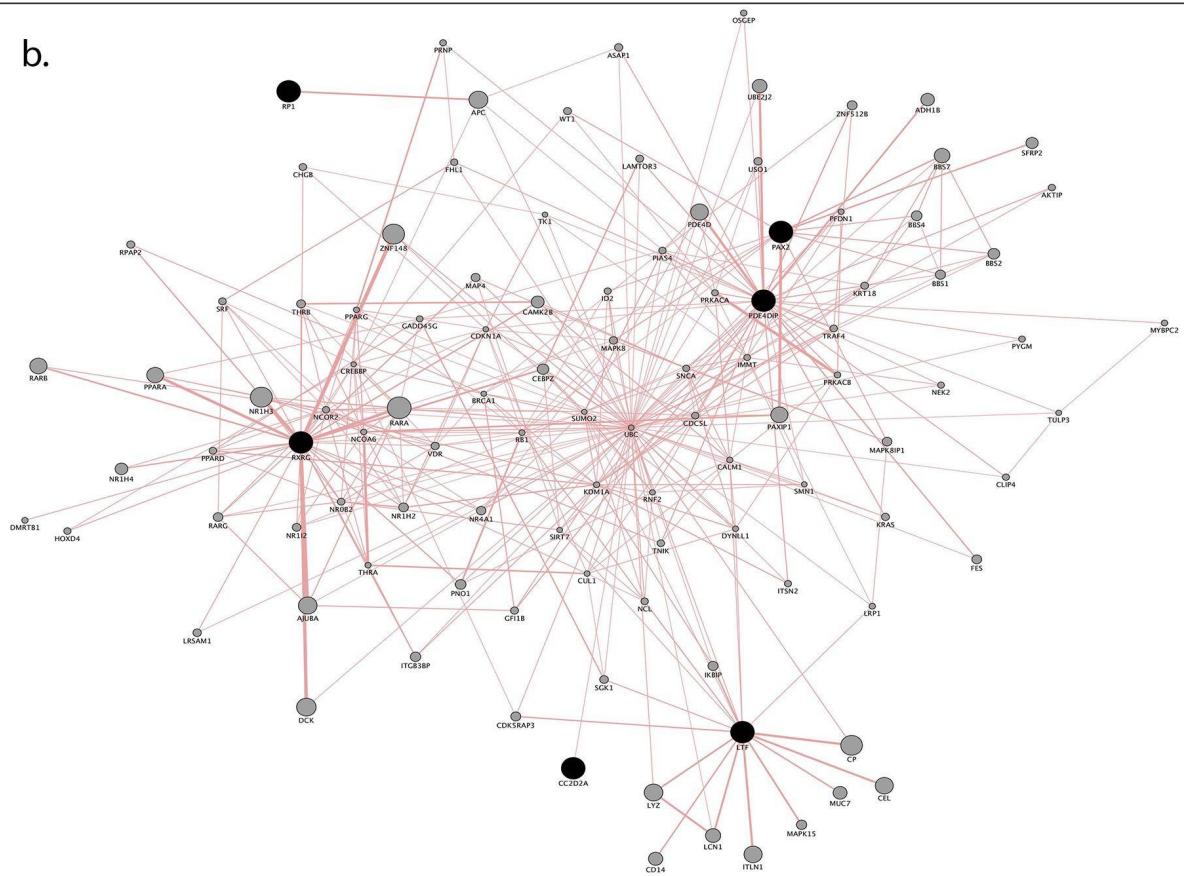

S4 Fig

Supplement: S4 Fig — The figure shows the Cytoscape preliminary pathway analysis, supported by GeneMANIA plug-in, with nodes and edges reflecting genetic (a) and physical (b) relationships between query genes. CACNG8 appears as the most genetic perturbing gene, while RXRG and PDE4DIP emerge as the most physical interacting genes. (PDF) [file pone.0278857.s004.pdf]

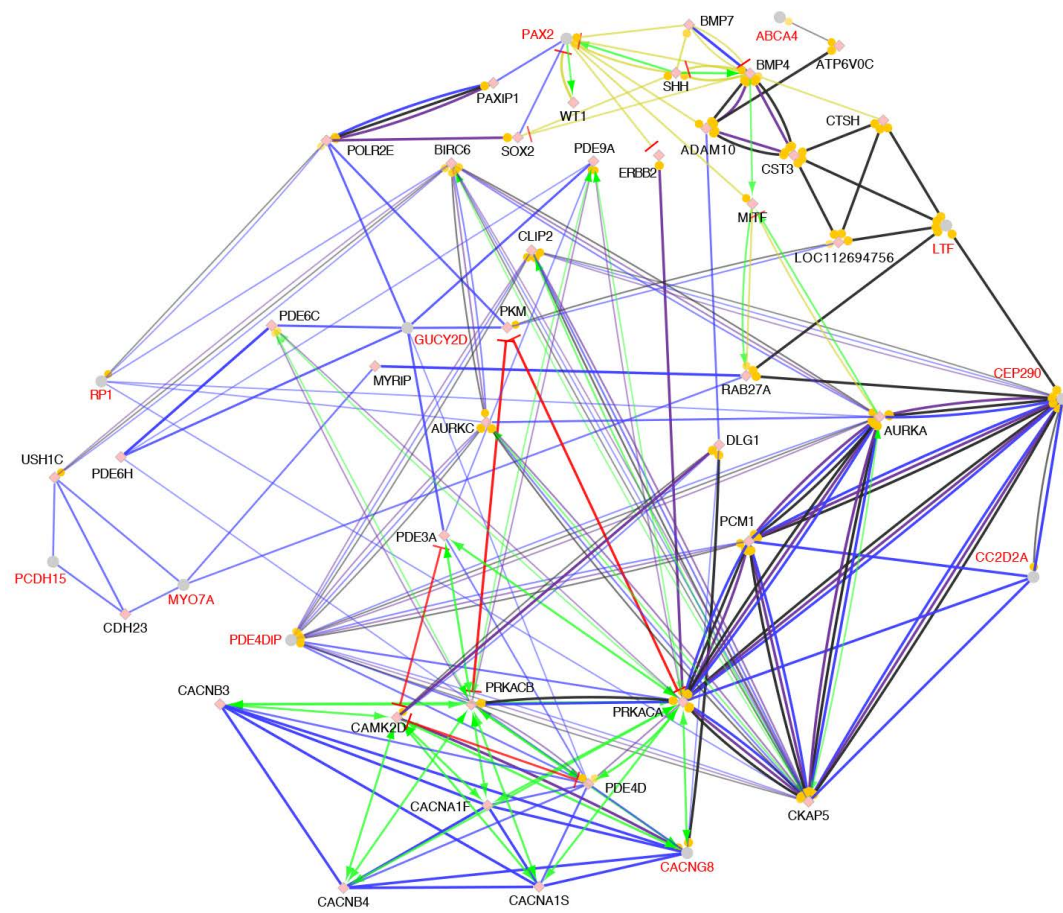

**S5 Fig**

Supplement: S5 Fig — Based on publicly available data from STRING and mIRANDA, 93100 terms were connected by 247 edges, related to enrichment categories: activation (green), binding (blue), catalysis (deep purple), expression (yellow), inhibition (red), ptmod (light purple), reaction (black). Only relevant enrichment (p < 0.05) in the identified protein interactome is shown. (PDF) [file pone.0278857.s005.pdf]

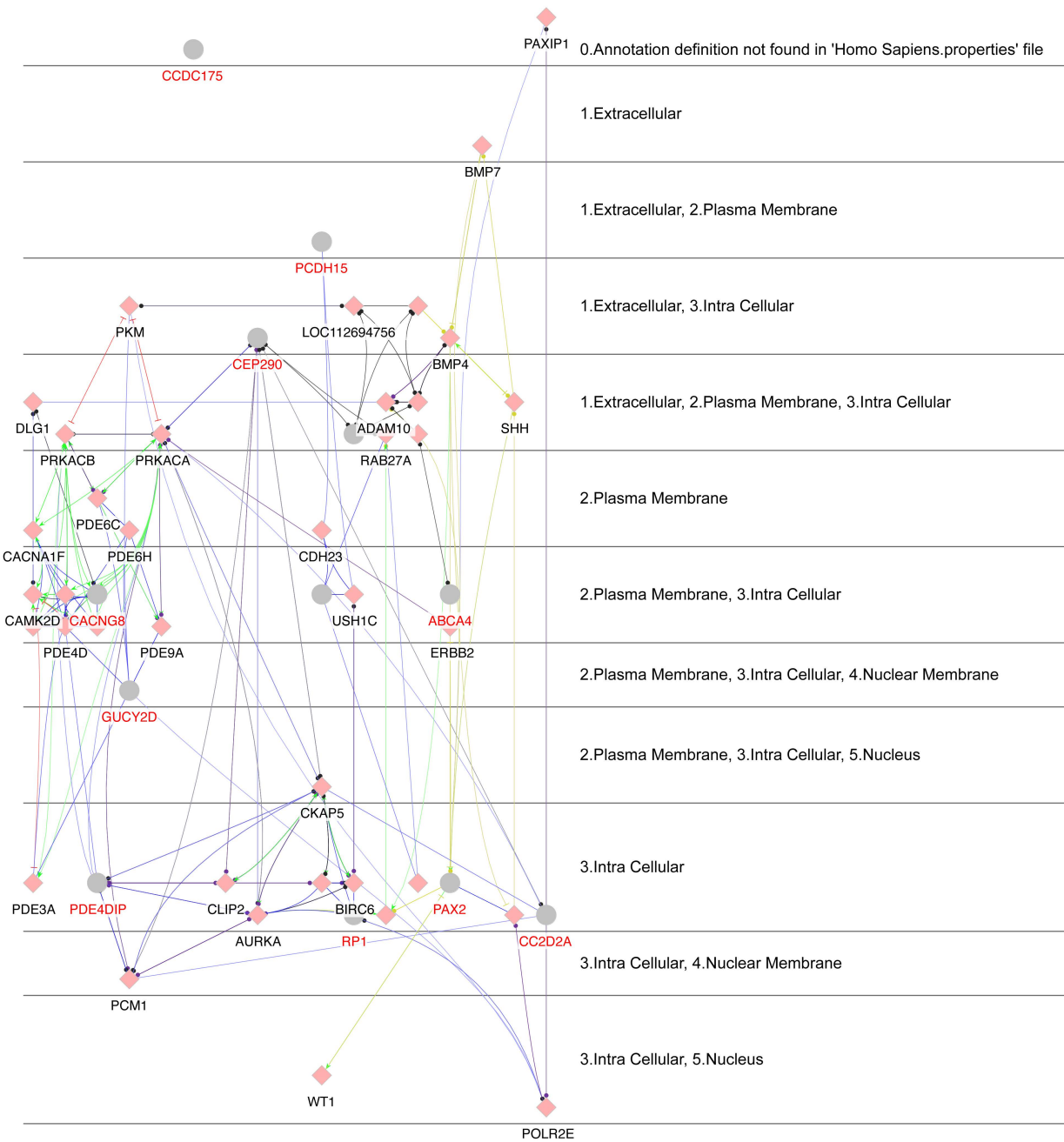

**S6 Fig**

Supplement: S6 Fig — Most of the proteins encoded by the 6 candidate genes are located in intracellular compartments with only one (LTF) extracellular and one (CACNG8) located in the plasma membrane. (PDF) [file pone.0278857.s006.pdf]

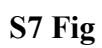

Supplement: S7 Fig — mIRANDA database showed that 2–9 miRNAs could regulate the expression of each analyzed gene. (PDF) [file pone.0278857.s007.pdf]

**a) GENOTYPES - CORR.**

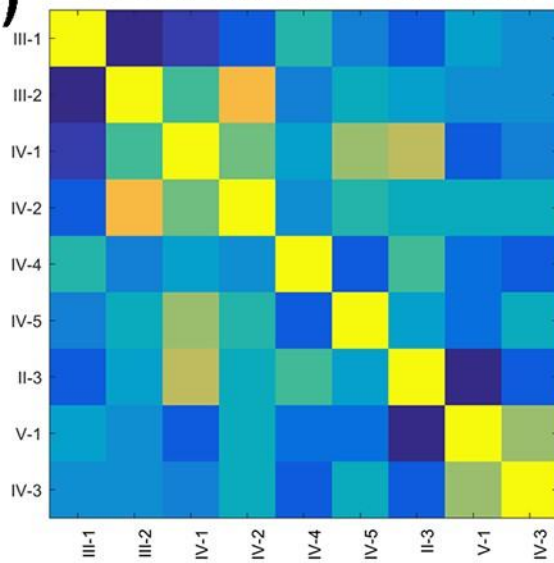

**b) GENOTYPES - 70% CUTOFF**

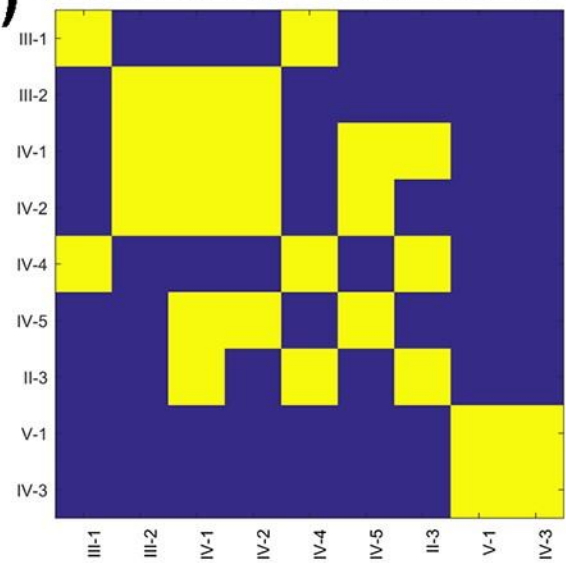

**c) SYMPTOMS - CORR.**

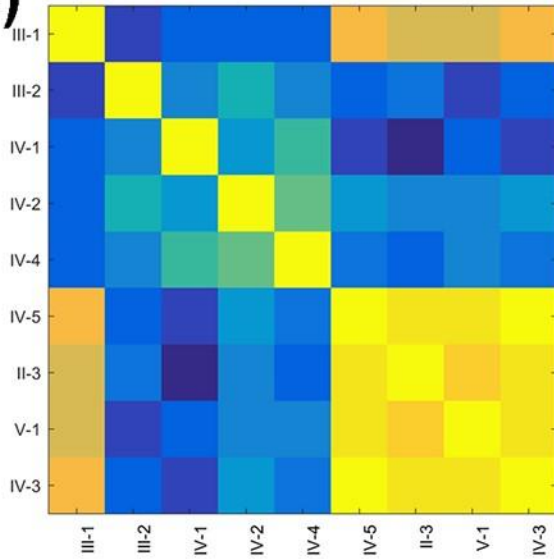

**d) SYMPTOMS - 80% CUTOFF**

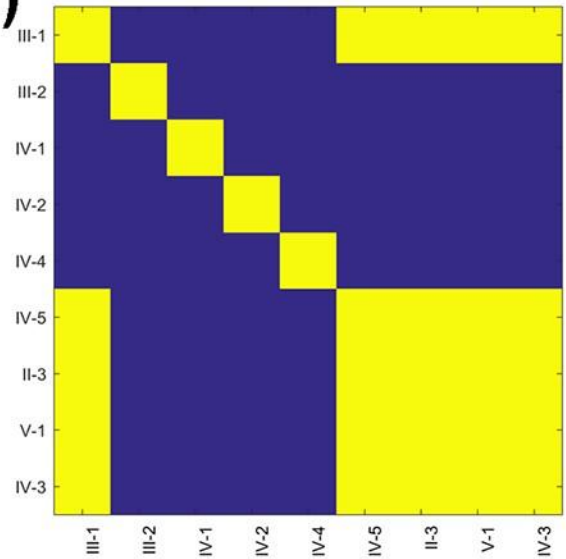

**S9 Fig**

Supplement: S9 Fig — a) Genotypes distribution of each subject was compared with those of all the other subjects. Such analysis resulted in a score ranging between 0 (blue) and 1 (yellow); the higher this value, the more similar genotypes distribution between two given subjects. b) By applying a cutoff to such maps at a given percentage, only pixels corresponding to subject pairs whose genotype distributions were more similar persist; here a representative similarity cutoff of 70% was chosen. c) The same correspondence could be obtained by considering symptom patterns. After applying a strong cutoff (80% in this case), subjects showing a close correspondence in symptoms distribution are highlighted (d). (PDF) [file pone.0278857.s009.pdf]

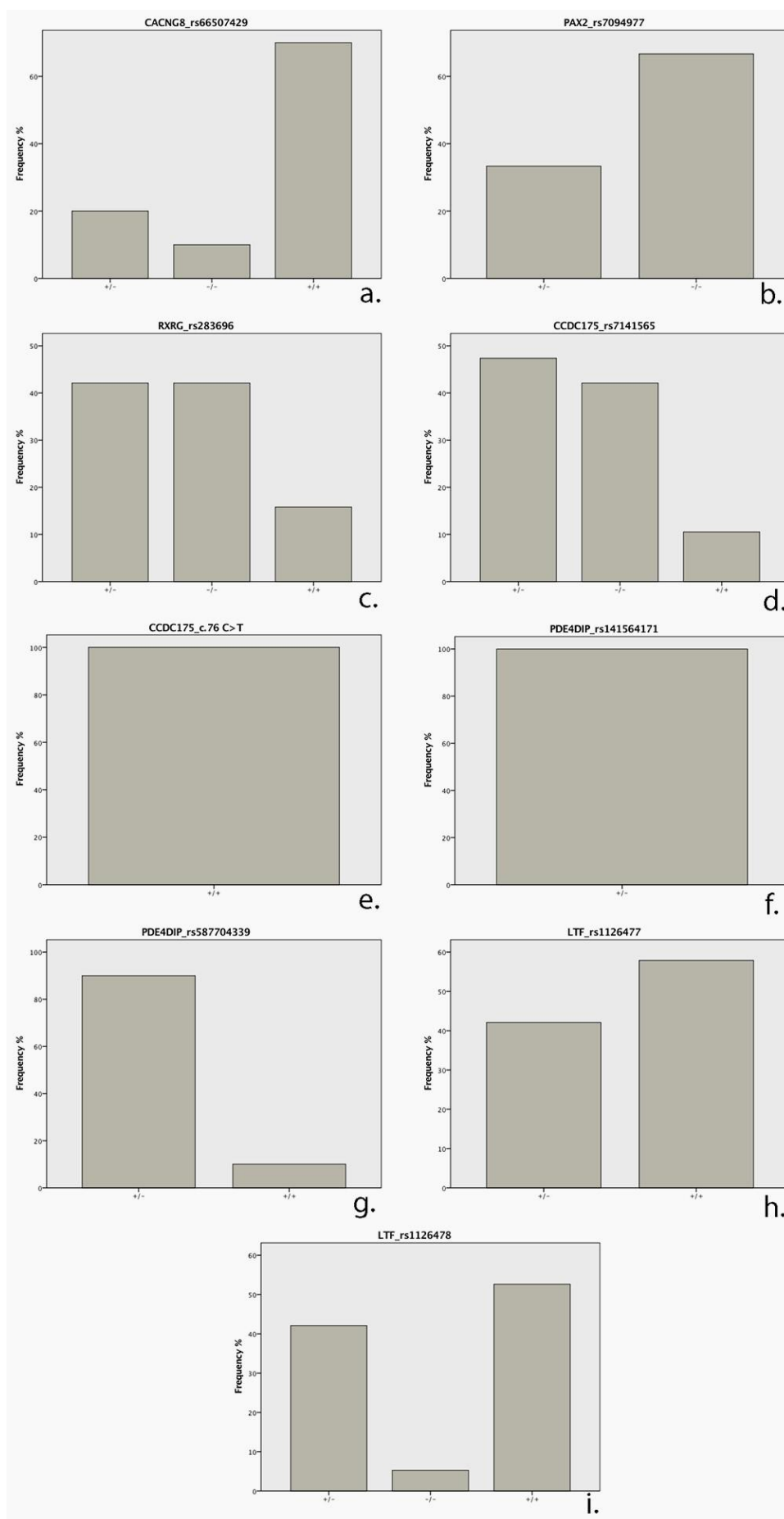

**S10 Fig**

Supplement: S10 Fig — As evidenced by bar plots (a-i), at least two variants, CACNG8 rs66507429:A>T (a) and CCDC175c.76 C>T (e), show a very low frequency distribution in healthy population, suggesting a possible association with pathology. (PDF) [file pone.0278857.s010.pdf]
