# Supplementary material for: The impact of modifier genes on cone-rod dystrophy heterogeneity: An explorative familial pilot study and a hypothesis on neurotransmission impairment
Source: PLoS One. 2022 Dec 9;17(12):e0278857. doi: 10.1371/journal.pone.0278857 (PMC9733859; doi:10.1371/journal.pone.0278857)
Supplement: S8 Fig — For each subject, genotypes of all analyzed variants are shown on a row of three heatmaps that are built according to an additive (a), dominant (b), or recessive (c) model, respectively. Each column thus represents the genotype of a given variant. In the additive model (a), yellow pixels correspond to wildtype, red ones to heterozygous mutation, and green pixels show mutations in homozygosis. In the dominant (b) and recessive models (c), red pixels highlight variants which were not considered as being mutated, whereas blue ones mean that, under the model considered, mutation occurred. (PDF) [file pone.0278857.s008.pdf]

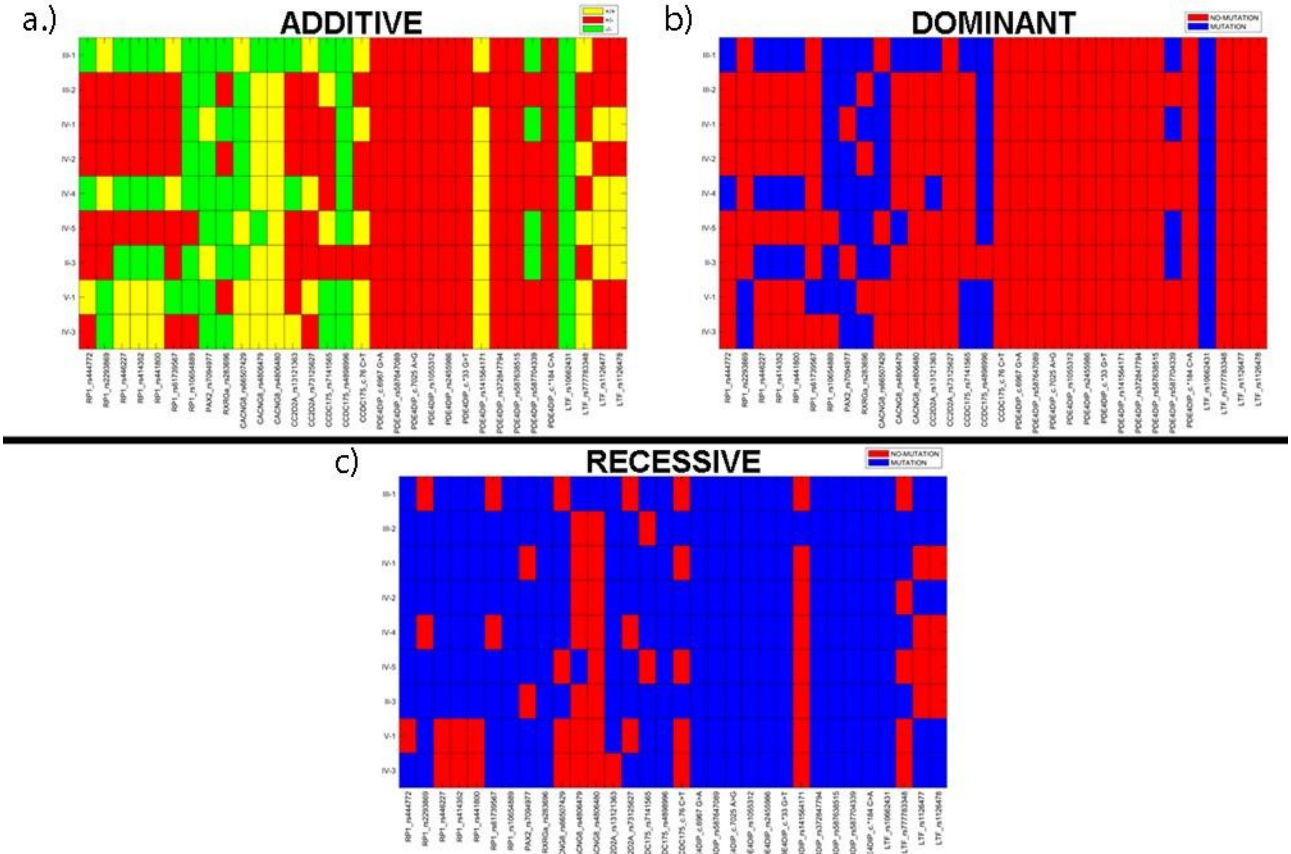
a.)
ADDITIVE
b)
DOMINANT
NO-MUTATION
MUTATION
c)
RECESSIVE
NO-MUTATION
MUTATION

S8 Fig
